# Amenability of coarse spaces and $\mathbb {K}$-algebras

**DOI:** 10.1007/s13373-017-0109-6

**Published:** 2017-11-09

**Authors:** Pere Ara, Kang Li, Fernando Lledó, Jianchao Wu

**Affiliations:** 1 Department of Mathematics, Universitat Autònoma de Barcelona, 08193 Bellaterra, Barcelona, Spain; 2 Department of Mathematics, University of Münster, Einsteinstr. 62, 48149 Münster, Germany; 3 Departamento de Matemáticas, Universidad Carlos III de Madrid, Avda. de la Universidad 30, 28911 Madrid, Spain; 4 Instituto de Ciencias Matemáticas (CSIC-UAM-UC3M-UCM), c./Nicolás Cabrera 13-15, Campus Cantoblanco UAM, Madrid, 28049 Spain; 5 Department of Mathematics, Penn State University, 109 McAllister Building, University Park, PA 16802 USA

**Keywords:** Amenability, Paradoxical decompositions, Følner nets, Coarse spaces, Unital $\mathbb {K}$-algebras, Leavitt path algebras, Translation algebras, 16P90, 43A07, 37A15, 20F65, 16S99

## Abstract

In this article we analyze the notions of amenability and paradoxical decomposition from an algebraic perspective. We consider this dichotomy for locally finite extended metric spaces and for general algebras over fields. In the context of algebras we also study the relation of amenability with proper infiniteness. We apply our general analysis to two important classes of algebras: the unital Leavitt path algebras and the translation algebras on locally finite extended metric spaces. In particular, we show that the amenability of a metric space is equivalent to the algebraic amenability of the corresponding translation algebra.

## Introduction

Given a group $\Gamma$, von Neumann defined in Section 1 of [53] the notion of *allgemeiner Mittelwert auf* $\Gamma$ in terms of a mean (i.e., a finitely additive probability measure) on $\Gamma$ which is left invariant under the action of $\Gamma$ on itself. This property of the group eventually came to be called amenability [27]. Its absence was recognized by von Neumann as a fundamental reason behind phenomena like the Banach–Tarski paradox—a paradoxical decomposition of the unit ball in $\mathbb {R}^3$. In fact, there is, for any group $\Gamma$, a complete dichotomy between amenability and the existence of paradoxical decompositions of $\Gamma$ in a natural sense, and the Banach–Tarski paradox may be essentially attributed to the fact that the (discrete) group SO(3) of isometries of the ball contains a subgroup which is isomorphic to the free group $\mathbb {F}_2$ on two generators, whose evident paradoxicality implies that of the former. By contrast, the group SO(2) of isometries of the unit disc, like any other abelian group, is amenable, and thus not paradoxical. Later, Følner gave an equivalent characterization of amenability by the existence of a net $\{\Gamma _i\}_{i \in I}$ of non-empty finite subsets of the group that, under the left translations of the group on itself, becomes more and more invariant in a statistical sense (cf., [37]). More precisely, one has that $\Gamma$ is amenable if and only if there exists a net $\{\Gamma _i\}_{i \in I}$ of non-empty finite subsets with

$$\begin{aligned} \lim _i\frac{|\gamma \Gamma _i \cup \Gamma _i|}{|\Gamma _i|}=1, \quad \text {for any } \gamma \in \Gamma , \end{aligned}$$

where $|\cdot |$ denotes the cardinality of the subset. These so-called Følner nets thus provide a good way to approximate an amenable infinite structure with finite substructures, opening the door to a wide range of applications. Moreover, thanks to its simplicity, Følner’s characterization also lends itself to various generalizations, as we shall see below. Since then, the concept of amenability has become central in many areas of mathematics like ergodic theory, geometry, the theory of operator algebras, etc. Some classical references on this topic are [47, 51, 54].

This paper studies amenability and paradoxical decompositions from an algebraic perspective. To provide a source of inspiration, we start with a review of amenability for metric spaces, a concept defined by Block and Weinberger in [19] through a natural generalization of Følner’s characterization to (uniformly) locally finite metric spaces—similar ideas go as far back as the work of Ahlfors ([4, II]) under the term *Ausschöpfungen einer offenen Fläche (exhaustions of an open surface)*. More precisely, a locally finite metric space (*X*, *d*) is said to be amenable if there exists a net $\{F_i\}_{i\in I}$ of finite non-empty subsets such that

$$\begin{aligned} \lim _{i} \frac{| N_R F_i |}{|F_i|} = 1, \quad \text {for any } R > 0, \end{aligned}$$

where $N_R F_i:= \{x\in X:d(x,F_i)\le R\}$, the *R*-neighborhood of $F_i$ (cf., Definition 2.1 and Remark 2.2). One of the key results in this setting, shown by Cecherini-Silberstein, Grigorchuk and de la Harpe in [23], states that in analogy with the well-known result for groups, the amenability of a metric space is equivalent to its non-paradoxicality, and also equivalent to the existence of an invariant mean, in a suitable sense (cf., Definitions 2.8 and 2.9). For the convenience of the reader, we present a direct proof of the most interesting implication among them, namely that non-paradoxicality implies amenability, by adapting a proof in the group setting given in [40] (cf., Theorem 2.17). The key idea in it is a local-to-global technique that involves a variant of Hall’s marriage theorem for sets of arbitrary cardinalities. A linearization of this technique will be applied later to prove a corresponding implication in the case of algebras over a field, which is the second main object of study in this article.

Let us fix a field $\mathbb {K}$. Elek introduced in [32] the notion of amenability for finitely generated unital algebras over $\mathbb {K}$, and proved some essential results in the case where the algebra has no zero-divisors. The main definition he used also resembles Følner’s characterization, with subsets replaced by linear subspaces, and cardinalities replaced by dimensions. We generalize this notion to $\mathbb {K}$-algebras of arbitrary dimensions and single out a more restrictive situation brought about by the additional requirement that the Følner net is exhaustive, which we term *proper amenability*.

### Definition 1

(*cf., Definition* 3.1 *and Remark* 3.2) Let $\mathbb {K}$ be a field. An $\mathbb {K}$-algebra ${\mathcal A}$ is said to be (left) *algebraically amenable* if there exists a net $\{W_i\}_{i\in I}$ of finite-$\mathbb {K}$-dimensional linear subspaces of ${\mathcal A}$ such that

$$\begin{aligned} \lim _{i} \frac{\dim _{\mathbb {K}}(a W_i +W_i)}{\dim _{\mathbb {K}}(W_i)} = 1, \quad \text {for any } a\in {\mathcal A}. \end{aligned}$$

If the net $\{W_i\}_{i\in I}$ can be chosen to satisfy the additional condition that for any $a \in {\mathcal A}$, there is $i\in I$ such that

$$\begin{aligned} a \in \bigcap _{j \ge i} W_j, \end{aligned}$$

then ${\mathcal A}$ is said to be (left) *properly algebraically amenable*.

Following Elek’s pioneering work, a number of authors have dealt with amenability for algebras from different perspectives, such as Bartholdi [15], Cecherini-Silberstein and Saimet-Vaillant [25], and D’Adderio [26] (building on work of Gromov [39]). Special attention has been paid by Elek to the case of division algebras over a field, see [30, 33, 34]. In particular, the notion of amenability for division algebras plays an important role in the study of infinite dimensional representations of a finite-dimensional algebra over a finite field undertaken in [30].

The fundamental result of Elek in [32] is the equivalence, for finitely generated unital $\mathbb {K}$-algebras without zero-divisors, among three characterizations of algebraic amenability analogous to those in the cases of groups and metric spaces: algebraic amenability à la Følner as given in Definition 1, the non-existence of paradoxical decompositions, and an analogue of von Neumann’s invariant means called invariant *dimension measures*. The definitions of the latter two notions enlist the involvement of linear bases of the algebra. We offer here generalizations of these notions (cf., Definitions 4.1 and 4.5) and of Elek’s theorem to encompass all $\mathbb {K}$-algebras regardless of the size of the generating set or the existence of zero-divisors or a unit. Notably, invariant dimension measures in our definition exhibit delicate deviations from von Neumann’s invariant means on a group, owing to the fact that the lattice of subspaces of an algebra is not distributive, unlike the lattice of subsets of a group. For the sake of brevity, here we state the generalized theorem only for countably dimensional $\mathbb {K}$-algebras.

### Theorem 2

(cf., Theorem 4.6 and Corollary 4.7) Let ${\mathcal A}$ be a countably dimensional $\mathbb {K}$-algebra over a field $\mathbb {K}$. Then the following are equivalent:

1. ${\mathcal A}$ is algebraically amenable.
2. There is a linear basis of ${\mathcal A}$ that cannot be paradoxically decomposed.
3. There exists an invariant dimension-measure on ${\mathcal A}$ associated to some linear basis.

By removing the requirement of finite generation, unitality, and having no zero-divisor, we can greatly expand the scope of examples subject to the study of amenability. Of foremost interest to us in this paper are two classes of algebras associated to geometric data:

1. *Leavitt path algebras* constructed from directed graphs (Definition 5.6): These algebras were introduced in [3] and [11] as generalizations of the classical algebras studied by Leavitt in [42, 43]. They also provide natural purely algebraic analogues of the widely studied graph $C^*$-algebras (see e.g. [48]). The class of Leavitt path algebras has interesting connections with various branches of mathematics, such as representation theory, ring theory, group theory, and dynamical systems. We refer the reader to [2] for a recent survey on this topic.
2. *Translation algebras* constructed from (locally finite) metric spaces (Definition 6.1):
  These algebras were introduced by Roe as an intermediate step between coarse metric spaces and a class of $C^*$-algebras now known as the (uniform) Roe $C^*$-algebras, as part of his far-reaching work on coarse geometry and the index theory for noncompact manifolds and metric spaces (cf., [49]). Their geometric nature enable them to serve as an important bridge between coarse geometry and the field of operator algebras, as well as a rich source of examples. We will further explore their connections to the theory of $C^*$-algebras in relation to amenability-type properties in [7].

Typically speaking, these algebras carry zero-divisors, and the translation algebras even have uncountable dimensions.

As corollaries of Theorem 2, we observe that *properly infinite* unital algebras are always non-amenable. Recall that a unital algebra ${\mathcal A}$ is said to be properly infinite if the unit is Murray–von Neumann equivalent to two mutually orthogonal idempotents. This condition itself expresses a form of paradoxicality, one that is generally strictly stronger than the notion of paradoxical decompositions used in Theorem 2. This Murray–von Neumann kind of paradoxical decomposition, along with some other forms of non-amenability, are discussed in [24, Section 4.5]. Indeed, there are division algebras which are non-amenable, and a division algebra cannot be properly infinite (cf. [33]). However, proper infiniteness and algebraic non-amenability coincide for the two main classes of examples we study.

### Theorem 3

(cf., Corollary 5.11 and Theorem 6.3) Let $\mathbb {K}$ be a field. If ${\mathcal A}$ is either

1. a unital Leavitt path $\mathbb {K}$-algebra of a finite graph, or
2. a translation $\mathbb {K}$-algebra (associated to a locally finite extended metric space),

then ${\mathcal A}$ is algebraically amenable if and only if it is not properly infinite.

In fact, in both cases, we pinpoint the necessary and sufficient properties of the underlying geometric data that give rise to the algebraic amenability of these algebras (cf., Theorems 5 and 5.10).

One novel aspect of our treatment is the careful distinction, in both the geometric setting and the algebraic setting, between the notion of amenability and the somewhat more restrictive notion of proper amenability, which, as described in Definition 1, asks for a Følner net that is exhaustive. In the group case as well as the case of ordinary metric spaces, these two concepts coincide (Corollary 2.19). However, subtle differences emerge once we engage *extended* metric spaces, that is, we allow the distance between two points to be infinite. A typical way for this to happen is for an infinite space to admit a finite *coarse connected component* (i.e., a finite cluster of points having finite distances among each other but infinite distances to the rest of the space), as this finite subset would immediately constitute a Følner net by itself, which is enough to witness amenability but not enough for proper amenability. In this sense, proper amenability ignores any Følner net that comes cheaply from an “isolated finite substructure”. It turns out such a typical way is, in fact, the only way to separate the two notions in this context (Corollary 2.20). In the algebraic setting, the distinction between the two concepts appears more pronounced, as they possess somewhat different permanence properties (cf., Proposition 3.6, Example 3.7 and Proposition 3.8). Nevertheless, we show that the disagreement between the two notions is always caused by the existence of a finite-dimensional (one-sided) ideal—again a prototypical “isolated finite substructure” in the relevant setting.

### Theorem 4

(cf., Theorem 3.9) Let ${\mathcal A}$ be an infinite dimensional $\mathbb {K}$-algebra over a field $\mathbb {K}$ that is algebraically amenable but not properly algebraically amenable. Then ${\mathcal A}$ has a finite-dimensional left ideal.

It follows from this theorem that algebraic amenability and proper algebraic amenability also agree for algebras without zero-divisors.^1^ The distinction between the two concepts eventually plays a role in the aforementioned generalization of Elek’s result in Theorem 2, even though the statement of the theorem does not mention proper algebraic amenability.

Although we only focus on the algebraic and the coarse geometric aspects of amenability in the present article, a major underlying motivation comes from their connections to the Følner property in the context of operator algebras. Such connections will be explored in [7], where we will investigate the close relationship between algebraic amenability and the existence of Følner nets of projections for operator algebras on a Hilbert space. We remark that Følner nets of projections are relevant in single operator theory [45], operator algebras (see, e.g., [9, 12, 18, 18]) as well as in applications to spectral approximation problems (see, e.g., [14, 20, 44] and references cited therein).

We conclude the article with some results connecting the two main objects of study in the paper—locally finite (extended) metric spaces and algebras over a field—through precisely the construction of the translation algebra of a locally finite (extended) metric space. With the help of the equivalent characterizations of amenability in both contexts, we obtain the satisfactory result that (proper) amenability of the metric space is equivalent to (proper) algebraic amenability of the corresponding translation algebra.

### Theorem 5

(cf., Theorems 6.3 and 6.4) Let (*X*, *d*) be a locally finite extended metric space and let $\mathbb {K}_\mathrm {u}(X)$ be its translation $\mathbb {K}$-algebra of a field $\mathbb {K}$. Then (*X*, *d*) is amenable (respectively, properly amenable) if and only if $\mathbb {K}_\mathrm {u}(X)$ is algebraically amenable (respectively, properly algebraically amenable).

In the case where the field $\mathbb {K}$ is the complex numbers $\mathbb {C}$, suitable completions of the translation algebras, the so-called *uniform Roe* $C^*$ *-algebras*, will be considered in [7], where further equivalences involving the Følner property of these $C^*$-algebras will be established.

*Contents* The paper is organized as follows. In Sect. 2, we begin by addressing the notion of amenability for locally finite extended metric spaces. We will recall in this context the relation to paradoxical decompositions and existence of invariant means in Theorem 2.11. Finally, we will completely clarify the relation between amenability and proper amenability for extended metric spaces in Sect. 2.1.

In Sect. 3, we analyze amenability issues in the context of algebras over a field $\mathbb {K}$, and give a complete analysis of the difference between algebraic amenability and proper algebraic amenability (see Proposition 3.6 and Theorem 3.9). If the $\mathbb {K}$-algebra has no zero-divisor, then algebraic amenability and proper algebraic amenability coincide (see Corollary 3.10).

Then we proceed in Sect. 4 to develop the relation between algebraic amenability, paradoxical decompositions and existence of dimension measures on the lattice of subspaces for general $\mathbb {K}$-algebras (i.e., not necessarily countably dimensional). This extends previous results by Elek in [32] in the context of countably dimensional algebras without zero-divisors. In this general setting, and due to the fact that the lattice of subspaces of an algebra is not distributive, the notion of additivity and invariance of dimension measures are captured by inequalities instead of equalities (see Definition 4.5 for details). Finally, we give examples of how to produce algebras that are not algebraically amenable using the dimension measure.

In the last two sections, we apply our general theory to two vast classes of examples: the Leavitt path algebras and the translation algebras. In Sect. 5, we prove that algebraic non-amenability and proper infiniteness coincide for the class of all unital Leavitt path algebras (see Theorem 5.10). Using the construction of path algebras, we also give simple examples where left and right algebraic amenability differ from each other. In Sect. 6, we prove the same result for the class of translation algebras associated to locally finite extended metric spaces. In fact, we also establish equivalences between the algebraic amenability of the translation algebra and the amenability of the underlying metric space (see Theorem 6.3), and the analogous equivalence for proper amenability (see Theorem 6.4).

*Notations* Given sets $X_1,X_2$ we write their cardinality by $|X_i|$, $i=1,2$ and their disjoint union by $X_1\sqcup X_2$. We put $\mathbb {N}_0=\{0,1,2,\ldots \}=\mathbb {N}\sqcup \{0\}$.

## Amenable metric spaces

In this section we will study locally finite metric spaces from a large scale geometric point of view. There are many interesting examples, of which the most prominent is the case of a finitely generated discrete group endowed with the word length metric. More generally, one can always equip any (countable) discrete group with a right- (or left-)invariant proper metric and obtain a metric space. The dependence on the right-invariant proper metric is a rather mild one, if one is only interested in the “large-scale” behavior of the metric space. More precisely, different right-invariant proper metrics on the same group induce metric spaces that are *coarsely equivalent*, see, e.g., Section 1.4 in [46]. Many important properties of groups are “large-scale” in nature. Examples include amenability, exactness, Gromov hyperbolicity, etc. In this section, we will focus on the first property in this list. Amenability has been well studied in coarse geometry (see, e.g., [46] or [21, Section 5.5]), so we will only emphasize the aspects which are important for establishing parallelism with the algebraic amenability for $\mathbb {K}$-algebras that we are going to investigate in the next sections. For the sake of simplicity, we will focus on *locally finite* metric spaces, i.e., those where any bounded set has finite cardinality.^2^

We start by recalling the definition of amenability for locally finite metric spaces. Our initial approach will make use of Følner sets. Let (*X*, *d*) be a metric space and *A* be a subset of *X*. For any $R>0$ define the following natural boundaries of *A*:

- *R*-*boundary:* $$\partial _R A:=\{x\in X: d(x,A)\le R\ \text {and}\ d(x,X{\setminus } A)\le R \}$$;
- *outer* *R* *-boundary:* $$\partial ^+_R A := \{x\in X{\setminus } A:d(x,A)\le R\}$$;
- *inner* *R* *-boundary:* $$\partial ^-_R A := \{x\in A:d(x,X{\setminus } A)\le R\}$$.

It is clear from the preceding definitions that $\partial _R A=\partial ^+_R A\sqcup \partial ^-_R A$. Next we introduce the notion of amenability of metric spaces due to Block and Weinberger (cf., [19, Section 3]).

### Definition 2.1

Let (*X*, *d*) be a locally finite metric space.

(i) Let $R>0$ and $\varepsilon \ge 0$. A finite non-empty set $F\subset X$ is called an $(R,\varepsilon )$-*Følner set* if it satisfies
  $$\begin{aligned} \frac{|\partial _R F|}{|F|}\le \varepsilon . \end{aligned}$$
  We denote by  the collection of $(R,\varepsilon )$-Følner sets.
(ii) The metric space (*X*, *d*) is called *amenable* if for every $R>0$ and $\varepsilon > 0$ there exists .
(iii) The metric space (*X*, *d*) is called *properly amenable* if for every $R>0$, $\varepsilon >0$ and finite subset $A\subset X$ there exists a  with $A\subset F$.

### Remark 2.2

Since with regard to the relation of set containment,  is monotonically decreasing with respect to *R* and monotonically increasing with respect to $\varepsilon$, we may also employ nets to simplify the quantifier-laden “local” condition used in the above definition:

(i) Amenability of (*X*, *d*) is equivalent to the existence of a net $\{F_i\}_{i\in I}$ of finite non-empty subsets such that
  $$\begin{aligned} \lim _{i} \frac{|\partial _R F_i|}{|F_i|} = 0, \quad \mathrm {for~all}\quad R > 0. \end{aligned}$$
(ii) Proper amenability of (*X*, *d*) requires, in addition, that this net $\{F_i\}_{i\in I}$ satisfies $X = \liminf _{i} F_i$, where $\liminf _{i} F_i := \bigcup _{j \in I} \bigcap _{i \ge j} F_i$.

### Example 2.3

For a finitely generated discrete group $\Gamma$ equipped with the word length metric both notions are equivalent to Følner’s condition for the group (see e.g., [46, Proposition 3.1.7]).

### Remark 2.4

With the convention that for any $x\in X$, $d(x,\varnothing )=\infty$, it is immediate that any finite set is properly amenable. Using the notation

$$\begin{aligned} N_R^+ A:= \{x\in X:d(x,A)\le R\} \quad \mathrm {and}\quad N_R^- A:= \{x\in X:d(x,X\setminus A) > R\}, \end{aligned}$$

we get the relations $\partial _R (N_{R}^+ A) \subset \partial ^+_{2R} A$ and $\partial _R (N_{R}^-A) \subset \partial ^-_{2R} A$. This shows that for both of the concepts of amenability in Definition 2.1, the use of the *R*-boundary may be replaced by either the outer or the inner *R*-boundary.

### Remark 2.5

From a coarse geometric point of view, the notion of (proper) amenability as defined above is better behaved when we restrict to metric spaces that are *uniformly locally finite* (some authors call them metric spaces with *bounded geometry*) in the sense that for any $R>0$, there is a uniform finite upper bound on the cardinalities of *all* closed balls with radius *R*, i.e.,

2.1 $$\begin{aligned} \sup _{x\in X}|B_R(x)|<\infty , \end{aligned}$$

where $B_R(x):=\{y\in X:d(x,y)\le R\}$ denotes the closed ball centered at *x* with radius *R*. The reason is that, for this class of metric spaces, amenability is preserved under coarse equivalence, and this gives us a natural way to generalize the definition to non-discrete metric spaces (satisfying a suitable notion of bounded geometry), c.f. [28, Proposition 3.D.32 and Definiton 3.D.33] or [19, Corollary 2.2 and Theorem 3.1]. This also holds true for proper amenability, with essentially the same argument (perhaps more easily seen with the aid of Lemma 2.6 below). However, for the results we are going to present, we generally do not require our metric space to be uniformly locally finite.

The following lemma shows that the definition of proper amenability can be already characterized in terms of the cardinality of the Følner sets.

### Lemma 2.6

Let (*X*, *d*) be an infinite locally finite metric space. Then *X* is properly amenable if and only if for every $R>0$, $\varepsilon >0$ and $N\in \mathbb {N}$ there exists an  such that $|F| \ge N$.

### Proof

The “*only if*” part is clear: for any $N\in \mathbb {N}$ just take a finite $A\subset X$ with $|A|=N$. To show the reverse implication let $R>0$, $\varepsilon >0$ and a finite $A\subset X$ be given. By assumption there is a finite $F\subset X$ such that

$$\begin{aligned} |F|\ge \frac{2 | \partial _R A|}{\varepsilon } \quad \mathrm {and}\quad \frac{|\partial _R F|}{|F|}\le \frac{\varepsilon }{2}. \end{aligned}$$

Putting $\widetilde{F}:=F\cup A$ (which contains *A*) we have

$$\begin{aligned} \frac{|\partial _R \widetilde{F}|}{|\widetilde{F}|} \le \frac{|\partial _R F|}{|F|} + \frac{|\partial _R A|}{|F|} \le \frac{\varepsilon }{2}+\frac{\varepsilon }{2}= \varepsilon \end{aligned}$$

and the proof is concluded. $\square$

As in the group case, the notion of amenability for metric spaces comes with an important dichotomy in relation to paradoxical decompositions. To formulate it, we first need to introduce an important tool in the study of coarse geometry.

### Definition 2.7

Let (*X*, *d*) be a locally finite metric space. A *partial translation on* *X* is a triple (*A*, *B*, *t*) consisting of two subsets *A* and *B* of *X* together with a bijection $t:A\rightarrow B$ such that the graph of *t* given by

$$\begin{aligned} \text {graph}(t):=\{(x,t(x))\in X\times X:x\in A\} \end{aligned}$$

is controlled, i.e., $\sup _{x\in A}d(x,t(x))<\infty$. We denote the corresponding domain and range of *t* by $\mathrm {dom}(t):=A$ and $\mathrm {ran}(t):=B$.

The set of all partial translations of *X* is denoted as $\mathrm {PT}(X)$.

Note that $\mathrm {PT}(X)$ forms a subsemigroup of the inverse semigroup of partially defined bijective maps *X* (see, e.g., [36]). More explicitly, the composition of any two partial translations $t, t' \in \mathrm {PT}(X)$, denoted by $t \circ t'$, is defined to be the partial translation satisfying

$$\begin{aligned} \mathrm {dom}( t \circ t') = \left\{ x \in \mathrm {dom}(t') \;|\; t'(x) \in \mathrm {dom}(t) \right\} \end{aligned}$$

and $( t \circ t') (x) = t(t'(x))$ for any $x \in \mathrm {dom}( t \circ t')$. Note that the graph of $t \circ t'$ is also controlled since

$$\begin{aligned} \sup _{x\in \mathrm {dom}( t \circ t')}d\left( x, ( t \circ t')(x)\right) \le \sup _{x\in \mathrm {dom}(t')}d(x,t'(x)) + \sup _{x\in \mathrm {dom}(t)}d(x,t(x)) < \infty . \end{aligned}$$

### Definition 2.8

A mean $\mu$ on a locally finite metric space (*X*, *d*) is a normalized, finitely additive map on the set of all subsets of *X*, $\mu :\mathcal {P}(X)\rightarrow [0,1]$. The measure $\mu$ is called *invariant under partial translations* if $\mu (A)=\mu (B)$ for all partial translations (*A*, *B*, *t*).

### Definition 2.9

Let (*X*, *d*) be a locally finite metric space. A *paradoxical decomposition of* *X* is a (disjoint) partition $X= X_+ \sqcup X_-$ such that there exist two partial translations $t_i:X\rightarrow X_i$ for $i \in \{+, - \}$.

### Remark 2.10

Applying a Bernstein-Schröder-type argument, one may slightly weaken the condition of having a paradoxical decomposition: it suffices to assume that there are two disjoint (non-empty) subsets $X'_+, X'_- \subset X$ such that there exist partial translations $t'_i :X \rightarrow X'_i$ for $i \in \{+,-\}$. Here we do not require their union to be *X*, in contrast with Definition 2.9. Indeed, assume we can find $(X'_+, t'_+, X'_-, t'_-)$ as above. We may then write $X = X'_+ \sqcup X'_- \sqcup \widetilde{X}$. Now we define $\widehat{X} = \bigcup \nolimits _{k=0}^\infty (t'_+)^k (\widetilde{X})$, where $(t'_+)^0$ is viewed as the identity map. This is a disjoint union because $\widetilde{X}$ is disjoint from the image of $t'_+$. Note also that $t'_+$ maps $\widehat{X}$ and $X{\setminus }\widehat{X}$ into themselves, respectively, and $\widehat{X} = \widetilde{X} \sqcup t'_+( \widehat{X} )$. By the injectivity of $t'_+$, we have $t'_+(X \setminus \widehat{X}) = X'_+ \setminus t'_+ (\widehat{X} ) = X'_+ \setminus \widehat{X}$. This allows us to construct a paradoxical decomposition $(X_+, t_+, X_2, t_2)$ in the sense of Definition 2.9 by setting $X_+ = X'_+ \sqcup \widetilde{X}$ (which is equal to $(X'_+ \setminus \widehat{X}) \sqcup \widehat{X}$), $X_2 = X'_-$, $t_+ = \left( t'_+ | _{X \setminus \widehat{X}} \right) \sqcup \mathrm {Id}_{\widehat{X}}$ and $t_2 = t'_-$.

The following result gives some standard characterizations of amenable metric spaces that will be used later (see, e.g., [23, Theorems 25 and 32]; we give an alternative proof of the implication (2)$\Rightarrow$(1) in the more general context of extended metric spaces; see in Theorem 2.17).

### Theorem 2.11

Let (*X*, *d*) be a locally finite metric space. Then the following conditions are equivalent:

1. (*X*, *d*) is amenable.
2. *X* admits no paradoxical decomposition.
3. There exists a mean $\mu$ on *X* which is invariant under partial translations.

### Remark 2.12

Deuber, Simonovits and Sós in [29] considered the exponential growth rate^3^ on locally finite metric spaces and they showed that this growth condition characterizes paradoxicality completely. It can be regarded as a Tarski-alternative-type theorem for locally finite metric spaces and it also served as an inspiration for the proof of the Tarski alternative (see [23, Theorem 32]).

It is interesting to note that the notions of paradoxicality and invariant means have been recently introduced and studied for arbitrary Boolean inverse monoids in [41].

### Amenability versus proper amenability for extended metric spaces

In many ways, the amenability for metric spaces generalizes the corresponding notion for groups, with certain properties paralleling those of the latter. However, caution should be taken when one tries to understand amenability for metric spaces from its similarity with groups. For example, amenability for metric spaces does not pass to subsets in general. As an example consider the free group $\mathbb {F}_n$, $n\ge 2$, with a ray attached to it. In this sense there is also a parallelism with the notion of Følner sequence in the context of operator algebras as considered in [9, Section 4].

In this subsection we complete the analysis of amenability in relation to proper amenability in the metric space context. We shall see that going beyond ordinary metric space (meaning the distance of any two points is finite) helps us better understand some aspects of amenability. For this we consider *extended* metric spaces (*X*, *d*) as coarse spaces, i.e., spaces where the metric is allowed to take the value $\infty$,

$$\begin{aligned} d:X\times X \rightarrow [0, \infty ]. \end{aligned}$$

For now let us stay assured that the additional complexity brought about by such a generalization is rather mild. Indeed, observe that the property that two points have finite distance defines an equivalence relation, which decomposes *X* uniquely into a disjoint union of equivalence classes $X= \bigsqcup _{i \in I} X_i$, such that each $(X_i, d|_{X_i \times X_i})$ is an ordinary metric space, while $d(X_i, X_j) = \infty$ for any different $i,j \in I$. Each $X_i$ is called a *coarse connected component* of *X*. Note that if (*X*, *d*) is a locally finite *extended* metric space, then each component $X_i$ is countable although the total space *X* need not be countable in general. As in the usual metric space situation we also have here that if *X* is finite, then it is properly amenable by taking $F=X$. As we will show later (Corollaries 2.19 and 2.20), it turns out that the notions of amenability and proper amenability are equivalent if the extended metric space contains only one coarse connected component (i.e., in the metric space case), but not in general.

#### Remark 2.13

Definitions 2.1, 2.7, 2.8 and 2.9 generalize directly to extended metric spaces. So does the Bernstein-Schröder-type argument in Remark 2.10.

#### Remark 2.14

We will justify here that the characterization of proper amenability in terms of the cardinality of the Følner sets given in Lemma 2.6 is still true in the extended metric space context. Note first that if $F\subset X=\bigsqcup _{i \in I} X_i$ is a finite set (and denoting by $F_i$ the corresponding subset in each coarse connected component $X_i$) we have that $d(x,F)=\min \{d(x,F_i) : i\in I\}$. Therefore, the *R*-boundary of *F* decomposes as *R*-boundaries in each coarse connected components:

$$\begin{aligned} \partial _R(F)=\mathop {\bigsqcup }_{i \in I} \partial _R (F_i). \end{aligned}$$

(Note also that if $F_i=\varnothing$, then $\partial _R (F_i)=\varnothing$). Therefore we can reason in each coarse connected component as in the proof of Lemma 2.6.

#### Proposition 2.15

Let (*X*, *d*) be a locally finite extended metric space. Then *X* is amenable if at least one of its coarse connected components is amenable. The converse is true in the case where there are only a finite number of coarse connected components.

#### Proof

The first statement is trivial. For the second, assume that $X = \bigsqcup _{i=1}^N X_i$ is a union of finitely many coarse connected components $X_i$, and that all the coarse connected components are non-amenable. We have to show that *X* is non-amenable. Since all coarse connected components $X_i$ are non-amenable, it follows from Theorem 2.11 that each component $X_i$ has a paradoxical decomposition. Since there is only a finite number of components, these paradoxical decompositions can be assembled to a paradoxical decomposition of *X*, hence *X* is non-amenable, as desired.$\square$

The second part of Proposition 2.15 cannot be generalized to extended metric spaces with an infinite number of coarse connected components, as the following example shows.

#### Example 2.16

We construct a locally finite extended metric space (*X*, *d*), with an infinite number of coarse connected components, such that neither of the connected components of *X* is amenable, but *X* is properly amenable. Let *Y* be the Cayley graph of the free non-Abelian group $\mathbb F _2$ of rank two. For each $n\in \mathbb {N}$, let $Y_n$ be the graph obtained by attaching *n* new vertices $v_1,\ldots , v_n$ and *n* new edges $e_1,\ldots , e_n$ to *Y*, in such a way that $e_i$ connects $v_i$ with $v_{i+1}$ for $i=1,\ldots ,n-1$, and $e_n$ connects $v_n$ with *e*, being *e* the neutral element of $\mathbb F_2$ (seen as a vertex of *Y*). Note that $Y_n$ is the graph obtained by attaching a trunk of length *n* to *Y*. Let $X_n$ be the metric space associated to the connected graph $Y_n$, and observe that all the metric spaces $X_n$ are non-amenable. Let *X* be the extended metric space having the metric spaces $X_n$ as coarse connected components. Then clearly *X* is properly amenable, because we can use the long trunks to localize the Følner sets of *X* of arbitrary large cardinality.

We also remark that Theorem 2.11 given in [23] stays true in the case of extended metric space.

#### Theorem 2.17

Let (*X*, *d*) be a locally finite extended metric space. Then the following conditions are equivalent:

1. (*X*, *d*) is amenable.
2. *X* admits no paradoxical decomposition.
3. There exists a mean $\mu$ on *X* which is invariant under partial translations.

#### Proof

The proofs of the implications (1) $\Rightarrow$ (3) and (3) $\Rightarrow$ (2) are standard and apply equally well to the extended metric space situation (see, e.g., [23, §26 and part III]).

The implication (2) $\Rightarrow$ (1) is more interesting. Hereby we present a direct proof for the sake of completeness, adapting ideas from Kerr and Li in [40, Theorem 3.4, (vi) $\Rightarrow$ (v)] to the setting of extended metric spaces (see also [40]). This proof should also serve as a motivation for the proof of Proposition 4.4 in the context of algebraic amenability.

We suppose that (*X*, *d*) is not amenable and would like to show that *X* has a paradoxical decomposition. By Remark 2.10, it suffices to show that there are two disjoint subsets $X'_+, X'_- \subset X$ such that there exist partial translations $t'_i :X \rightarrow X'_i$ for $i \in \{+,-\}$. By the negation of Definition 2.1, there is $\varepsilon _0 \in (0, 1)$ and $R_0>0$ such that, for any finite non-empty set $F\subset X$, one has the following estimate for the outer *R*-boundary: $|\partial ^+_{R_0} F|>\varepsilon _0 |F|$ and, hence, $|N^+_{R_0} F|>(1+\varepsilon _0) |F|$. Since, for any finite set $F\subset X$, we also have

$$\begin{aligned} N^+_{2R_0}(F)\ge N^+_{R_0}\left( N^+_{R_0} F \right) \ge (1+\varepsilon _0)| N^+_{R_0} F | \ge (1+\varepsilon _0)^2 | F |, \end{aligned}$$

we can choose a radius $R_d:=nR_0$ for some $n\ge \log _{1+\varepsilon _0}(2) +1$ satisfying the following local doubling condition: for any finite non-empty set $F\subset X$, we have

$$\begin{aligned} |N^+_{R_d} F | > 2 \;|F|. \end{aligned}$$

In the next step of the proof we will essentially use Zorn’s lemma to produce a paradoxical decomposition (a “global doubling”) of *X*. Consider the set $\Omega$ of set-valued maps $\omega :X\times \{ {\scriptstyle +,-}\}\rightarrow {\mathcal P}(X)$ (the power set of *X*) such that for any $y=(x,j)\in X\times \{{\scriptstyle +,-}\}$ we have $\omega (y)\in {\mathcal P}\left( B_{R_d}(x)\right)$ and for any finite set $K\subset X\times \{{\scriptstyle +,-} \}$ we have

$$\begin{aligned} \left| \mathop {\bigcup }_{y\in K} \omega (y) \right| \ge |K|. \end{aligned}$$

Note that the set $\Omega$ is not empty since the set-valued map given by $\omega (y):=B_{R_d}(x)$ for any $y=(x,j)\in X\times \{{\scriptstyle +,-} \}$ is an element of $\Omega$. In fact, we only need to verify the preceding inequality: for any finite set $K \subset X\times \{{\scriptstyle +,-} \}$, we write $K = K_+\times \{{\scriptstyle +}\}\sqcup K_-\times \{{\scriptstyle -}\}$ and calculate that

$$\begin{aligned} \left| \mathop {\bigcup }_{y\in K} \omega (y) \right| = \left| N_{R_d}^+(K_+\cup K_-) \right| \ge 2|K_+\cup K_-|\ge |K_+|+|K_-|=|K|. \end{aligned}$$

The set $\Omega$ may also be partially ordered in the following natural way

$$\begin{aligned} \omega \le \omega ' \quad \mathrm {if}\quad \omega (y)\subset \omega '(y)\quad \mathrm {for~any~} y\in X\times \{{\scriptstyle +,-}\}. \end{aligned}$$

Since any descending chain has a non-empty lower bound given by pointwise intersection we obtain by Zorn’s lemma a minimal element $\omega _m\in \Omega$. Note that, by the definition of $\Omega$, we already have $| \omega _m(y) |\ge 1$ for any $y\in X\times \{{\scriptstyle +,-}\}$.

We claim that $|\omega _m(y)|= 1$ for any $y\in X\times \{{\scriptstyle +,-}\}$. Suppose this is not the case. Then there is $y_0\in X\times \{{\scriptstyle +,-}\}$ such that $\omega _m(y_0)$ has two distinct elements $x_+,x_-$. By the minimality of $\omega _m$, there exist, for $l\in \{{\scriptstyle +,-}\}$, finite sets $K_l\subset X\times \{{\scriptstyle +,-}\}$ not containing $y_0$ and such that

$$\begin{aligned} \left| \left( \omega _m(y_0){\setminus }\{x_l\} \right) \cup \left( \mathop {\bigcup }_{y\in K_l} \omega _m (y)\right) \right| \le |K_l|. \end{aligned}$$

(Note that, otherwise, one could remove $x_l$ from $\omega _m(y_0)$ to specify a new element in $\Omega$ strictly smaller than $\omega _m$.) Define, for $l\in \{{\scriptstyle +,-}\}$, the set

$$\begin{aligned} Z_l:= \left( \omega _m(y_0){\setminus }\{x_l\} \right) \cup \left( \mathop {\bigcup }_{y\in K_l} \omega _m (y)\right) {.} \end{aligned}$$

Using the identity $(\omega _m(y_0){\setminus }\{x_+\})\cup (\omega _m(y_0){\setminus }\{x_-\})=\omega _m(y_0)$ as well as the preceding inequality, we obtain the following contradiction:

$$\begin{aligned} |K_+|+|K_-|\ge & {} |Z_+|+|Z_-| \;=\;|Z_+\cup Z_-|+|Z_+\cap Z_-| \\\ge & {} \left| \omega _m(y_0)\cup \left( \mathop {\bigcup }_{y\in (K_+\cup K_-)} \omega _m(y)\right) \right| + \left| \mathop {\bigcup }_{ y\in (K_+\cap K_-)} \omega _m(y) \right| \\\ge & {} 1+|K_+\cup K_-|+|K_+\cap K_-|= 1+|K_+|+|K_-|. \end{aligned}$$

Therefore $|\omega _m(y)|= 1$ for any $y\in X\times \{{\scriptstyle +,-}\}$.

To finish the proof, we define, for any $l\in \{{\scriptstyle +,-}\}$, the map $t_l:X\rightarrow X$ which assigns to each $x\in X$ the unique element in $\omega _m(x,l)$. Note that it follows now from the definition of $\Omega$ that $\omega _m(y) \cap \omega _m(y') = \varnothing$ if $y\not = y'$. Consequently, both $t_+$ and $t_-$ are injective and they have disjoint images, which we denote by $X_+$ and $X_-$, respectively. Since by definition $\omega _m(x,l)\subset B_{R_d}(x)$ we have

$$\begin{aligned} \sup \{d(x,t_l(x)) : x\in X\}\le R_d, \end{aligned}$$

hence the maps $t_+$, $t_-$ are controlled and the quadruple $(X_+, t_+, X_-, t_-)$ satisfies the condition in Remark 2.10 and, hence, a paradoxical decomposition can be obtained from them. $\square$

The next proposition is the key to our results on the relationship between amenability and proper amenability for extended metric spaces.

#### Proposition 2.18

Let (*X*, *d*) be a non-empty locally finite extended metric space, and assume that all the coarse connected components of *X* are infinite. Then *X* is amenable if and only if *X* is properly amenable.

#### Proof

Suppose that $X= \bigsqcup _{i\in I} X_i$ is amenable, where $X_i$ are the coarse connected components of *X*. By Remark 2.14, it is enough to check that for $R>0$ and $\varepsilon >0$ the sets in  have unbounded cardinality. Suppose this is not the case, i.e., there is $R_0>0$, $\varepsilon _0$ with $1>\varepsilon _0>0$ and $N_0\in \mathbb {N}$ such that  has an element $F_0$ of maximal cardinality $N_0$. Write $F_0= \bigsqcup _{i\in I_0} F_{0,i}$, where $F_{0,i}$, $i\in I_0$, are the (non-empty) coarse connected components of $F_0$, so that $F_{0,i}= F_0\cap X_i\ne \varnothing$ for $i\in I_0$, and $I_0$ is a finite subset of *I*. Set

$$\begin{aligned} R_1:= \mathrm {max}_{i\in I_0} \{ \mathrm {diam}(F_{0,i}) + \mathrm {dist}(F_{0,i}, X_i{{\setminus }}F_{0,i}) \} , \end{aligned}$$

where $\mathrm {diam}(F_{0,i})=\max {\{d(x,y) : x,y\in F_{0,i}\}}$ is the diameter of $F_{0,i}$. Observe that $X_i {\setminus } F_{0,i}$ is non-empty by our hypothesis that all the coarse connected components of *X* are infinite.

Choose $R>R_0+R_1$, and $\varepsilon >0$ such that

$$\begin{aligned} \varepsilon < \mathrm {min} \Big \{ \varepsilon _0, \frac{1}{|F_0|} \Big \}. \end{aligned}$$

Since *X* is amenable, there exists . We claim that $F\not \subset F_0$. Indeed, if $F\subset F_0$, then by the choice of $R_1$ we have $F_{0,i} \subset \partial _R F_i$ for all $i\in I_0$ such that the coarse connected component $F_i$ of *F* is non-empty. Let $I_0'$ be the (non-empty) subset of $I_0$ consisting of those $i\in I_0$ such that $F_i\ne \varnothing$. Then we obtain

$$\begin{aligned} \frac{|\partial _R F|}{|F|} \ge \frac{\sum _{i\in I_0'}|F_{0,i}|}{\sum _{i\in I_0'}|F_{0,i}|} = 1> \varepsilon _0 > \varepsilon , \end{aligned}$$

hence , proving our claim. Write $F=\bigsqcup _{j\in J_0} F_j$, where $J_0$ is finite, and $\{ F_j : j\in J_0 \}$ are the (non-empty) coarse connected components of *F*. It follows that for some coarse connected component $F_{j_0}$ of *F*, we have $F_{j_0}\not \subset F_{0}$.

For $k\in I_0\cup J_0$, set $F_{0,k} = F_0\cap X_k$ and $F_k= F\cap X_k$. (Note that some $F_{0,k}$ or some $F_k$ might be empty.)

We consider next two cases:

- If $$\partial _R(F) \ne \varnothing $$, then
  $$\begin{aligned} \frac{1}{|F|} \le \frac{|\partial _R(F)|}{|F|} \le \varepsilon <\frac{1}{|F_0|} \end{aligned}$$
  and so, $N_0= |F_0|< |F|$. Hence  with $|F|>N_0$, which is a contradiction to the maximality of $N_0$.
- If $\partial _R (F) = \varnothing$ we have two possibilities, for each $j\in J_0$:
  (i) If $F_j\cap F_{0, j}\not =\varnothing$, then $F_{0,j} \subset F_j$ by using our assumption that $\partial _R(F)=\varnothing$.
  (ii) $$F_j \cap F_{0,j}=\varnothing $$.
  Assume that condition (ii) holds for some $j_0\in J_0$. Then $\widetilde{F}:= F_0\sqcup F_{j_0}$ satisfies
  $$\begin{aligned} \frac{|\partial _{R_0} (\widetilde{F})|}{|\widetilde{F}|} \le \frac{|\partial _{R_0} (F_0)|+|\partial _{R_0} (F_{j_0})|}{|F_0|+|F_{j_0}|} = \frac{|\partial _{R_0} (F_0)|}{|F_0|+|F_{j_0}|} < \frac{|\partial _{R_0} (F_0)|}{|F_0|}\le \varepsilon _0 , \end{aligned}$$
  where the equality follows from the fact that $\partial _R (F) = \varnothing$. Thus $\widetilde{F}$ is a $(R_0,\varepsilon _0)$-Følner set with $| \widetilde{F}| >N_0$ and we have a contradiction. If case (i) occurs for all $j\in J_0$, then $J_0\subset I_0$ and $F_{0,j}\subset F_j$ for all $j\in J_0$. Writing $\widetilde{F} = F_0 \cup F$, we have that $|\widetilde{F}| >|F_0|= N_0$, because $F\not \subset F_0$. Setting $I_0'':= I_0{\setminus } J_0$, we get, using that $\partial _{R_0}F_j= \varnothing$ for all $j\in J_0$,
  $$\begin{aligned} \frac{|\partial _{R_0}\widetilde{F}|}{|\widetilde{F}|}&= \frac{\sum _{j\in J_0} |\partial _{R_0}F_j| + \sum _{i\in I_0''} |\partial _{R_0}F_{0,i}|}{|\widetilde{F}|}\\&= \frac{ \sum _{i\in I_0''} |\partial _{R_0}F_{0,i}|}{|\widetilde{F}|} \le \frac{|\partial _{R_0}F_0|}{|F_0|}\le \varepsilon _0, \end{aligned}$$
  so that $\widetilde{F}$ is a $(R_0,\varepsilon _0)$-Følner set of cardinality strictly larger than $N_0$, which is again a contradiction.

In either case we get a contradiction to the maximality of $N_0$ and the proof is concluded. $\square$

As an immediate consequence of Proposition 2.18, we obtain the following result.

#### Corollary 2.19

Let (*X*, *d*) be a locally finite metric space. Then (*X*, *d*) is amenable if and only if (*X*, *d*) is properly amenable.

We can now obtain the characterization of the amenable but not properly amenable extended metric spaces. This should be compared to Theorem 3.9 in the algebraic setting.

#### Corollary 2.20

Let (*X*, *d*) be a locally finite extended metric space with infinite cardinality. Then *X* is amenable but not properly amenable if and only if $X= Y_1\sqcup Y_2$, where $Y_1$ is a finite non-empty subset of *X*, $Y_2$ is non-amenable and $d(x,y)= \infty$ for $x\in Y_1$ and $y\in Y_2$.

#### Proof

Assume first that $X= Y_1\sqcup Y_2$, where $Y_1$ is a finite non-empty subset of *X*, $Y_2$ is non-amenable and $d(x,y)= \infty$ for $x\in Y_1$ and $y\in Y_2$. Observe that $Y_1$ is the disjoint union of some coarse connected components of *X*, and $Y_2$ is the disjoint union of the rest of the coarse connected components of *X*. Clearly $Y_1$ is a finite non-empty subset of *X* such that $\partial _R(Y_1)=\varnothing$ for all $R>0$. Hence *X* is amenable. One can easily show that, if *X* is properly amenable, then $Y_2$ is also properly amenable, contradicting our hypothesis. Indeed, given and $R>0$, $\varepsilon >0$ and $N>0$, take a subset *F* of $Y_2$ such that

$$\begin{aligned} \frac{|\partial _R(Y_1\sqcup F)|}{|Y_1\sqcup F|} \le \delta , \end{aligned}$$

where $\delta$ satisfies $0< \delta (1+\delta ) < \varepsilon$, and $|F|\ge \mathrm {max} \{N, \frac{|Y_1|}{\delta }\}$. Then *F* is a $(R,\epsilon )$-Følner subset of $Y_2$ with $|F|\ge N$, as desired. Hence, *X* is amenable but not properly amenable.

Suppose now that *X* is amenable but not properly amenable. We first show that there are only a finite number of finite components. Indeed, if $X_1,X_2,\ldots ,$ is an infinite sequence of finite coarse connected components, then $\bigsqcup _{i=1}^n X_i$ are Følner (*R*, 0)-subsets of unbounded cardinality in *X*, and so *X* is properly amenable by Remark 2.14, giving a contradiction. Hence there is only a finite number of finite coarse connected components $X_1,\ldots , X_N$. Let $Y_1= \bigsqcup _{i=1}^N X_i$, and let $Y_2=X{\setminus } Y_1$. Then all the coarse connected components of $Y_2$ are infinite. If $Y_2$ is amenable, then it is also properly amenable by Proposition  2.18, and so *X* is also properly amenable, contradicting our hypothesis. Hence $Y_2$ is non-amenable. Since *X* is amenable by hypothesis, we conclude that $Y_1\ne \varnothing$. This concludes the proof. $\square$

## Algebraic amenability

In this section we will analyze from different points of view a version of amenability for $\mathbb {K}$-algebras, where $\mathbb {K}$ is a field. Our definition will follow existing notions in the literature (see Section 1.11 in [38] and [25, 32]), but we aim to generalize previous definitions and results in a systematical fashion. To simplify terminology, we will often not mention $\mathbb {K}$ explicitly. For instance, we may call $\mathbb {K}$-algebras just algebras, and $\mathbb {K}$-dimensions just dimensions.

### Definition 3.1

Let ${\mathcal A}$ be a $\mathbb {K}$-algebra.

(i) Let $\mathcal {F}\subset {\mathcal A}$ be a finite subset and $\varepsilon \ge 0$. Then a nonzero finite-dimensional linear subspace $W \subset {\mathcal A}$ is called a left $(\mathcal {F}, \varepsilon )$ *-Følner subspace* if it satisfies
  3.1 $$\begin{aligned} \frac{\dim (a W +W)}{\dim (W)}\le 1+\varepsilon , \quad \mathrm {for~all}\quad a\in \mathcal {F}. \end{aligned}$$
  The collection of $(\mathcal {F}, \varepsilon )$-Følner subspaces of ${\mathcal A}$ is denoted by .
(ii) ${\mathcal A}$ is left *algebraically amenable* if for any $\varepsilon >0$ and any finite set $\mathcal {F}\subset {\mathcal A}$, there exists a left $(\mathcal {F}, \varepsilon )$-Følner subspace.
(iii) ${\mathcal A}$ is *properly* left algebraically amenable if for any $\varepsilon >0$ and any finite set $\mathcal {F}\subset {\mathcal A}$, there exists a left $(\mathcal {F}, \varepsilon )$-Følner subspace *W* such that $\mathcal {F} \subset W$.

We may also define *right* Følner subspaces, *right* algebraic amenability and proper *right* algebraic amenability by replacing ${\mathcal A}$ with ${\mathcal A}^\mathrm {op}$ in the above definitions. Since the two situations are completely symmetric, we will stick with the left versions of the definitions. For simplicity we are going to drop the term “left” for the rest of this section. Any algebra satisfying $\dim ({\mathcal A})<\infty$ is obviously properly algebraically amenable by taking $W={\mathcal A}$.

### Remark 3.2

There are some slightly different, but equivalent, ways to define (proper) algebraic amenability. For example, since for any $\varepsilon >0$ and any finite set $\mathcal {F}\subset {\mathcal A}$, an $(\mathcal {F}, \varepsilon )$-Følner subspace also satisfies

$$\begin{aligned} \frac{\dim ( \mathrm {span}( \mathcal {F} W +W))}{\dim (W)}\le 1+ |\mathcal {F}| \varepsilon , \end{aligned}$$

we may equivalently define algebraic amenability for ${\mathcal A}$ as saying that for any $\varepsilon >0$ and any finite set $\mathcal {F}\subset {\mathcal A}$, there exists a nonzero finite-dimensional linear subspace *W* such that

$$\begin{aligned} \frac{\dim ( \mathrm {span}( \mathcal {F} W +W))}{\dim (W)}\le 1+\varepsilon . \end{aligned}$$

Since with regard to the relation of set containment,  is monotonically decreasing with respect to $\mathcal {F}$ and monotonically increasing with respect to $\varepsilon$, we may also employ nets to simplify the quantifier-laden “local” condition used in the above definition:

(i) Algebraic amenability of ${\mathcal A}$ is equivalent to the existence of a net $\{W_i\}_{i\in I}$ of finite-dimensional linear subspaces such that
  $$\begin{aligned} \lim _{i} \frac{\dim (a W_i +W_i)}{\dim (W_i)} = 1, \quad \mathrm {for~all}\quad a\in {\mathcal A}. \end{aligned}$$
(ii) Proper algebraic amenability of ${\mathcal A}$ requires, in addition, that this net $\{W_i\}_{i\in I}$ satisfies ${\mathcal A}= \liminf _{i} W_i$, where $\liminf _{i} W_i := \bigcup _{j \in I} \bigcap _{i \ge j} W_i$.

### Remark 3.3

(i) The notion given by Elek in Definition 1.1 of [32] in fact corresponds to *proper* algebraic amenability, as will become evident in the next proposition (see also Definition 3.1 in [25]). Nevertheless, since the main results in Elek’s paper restrict to the case of algebras with no zero divisors, amenability and algebraic amenability are equivalent (see Corollary 3.10 below).
(ii) In Definition 4.3 of [15], Bartholdi uses the name *exhaustively amenable* instead of properly amenable.

Notice that although the definition works for $\mathbb K$-algebras of arbitrary dimensions, the property of algebraic amenability is in essence a property for countably dimensional algebras, as seen in the next proposition.

### Proposition 3.4

A $\mathbb K$-algebra ${\mathcal A}$ is (properly) algebraically amenable if and only if any countable subset in ${\mathcal A}$ is contained in a countably dimensional $\mathbb K$-subalgebra that is (properly) algebraically amenable.

### Proof

For the forward direction, we assume ${\mathcal A}$ is (properly) algebraically amenable and let $\mathcal C \subset {\mathcal A}$ be an arbitrary countable subset. Using the fact that a subalgebra generated by a countable set or a countably dimensional linear subspace is countably dimensional, we define an increasing sequence $\{{\mathcal B}_i\}_{i=0}^\infty$ of countably dimensional $\mathbb K$-subalgebras in ${\mathcal A}$ as follows:

- We let ${\mathcal B}_0$ be the subalgebra generated by $\mathcal C$.
- Suppose ${\mathcal B}_i$ has been defined. Let $\{e_k\}_{k=1}^\infty$ be a basis of ${\mathcal B}_i$. By the (proper) algebraic amenability of ${\mathcal A}$, for each positive integer *k*, we may find a finite dimensional linear subspace $W_k \subset {\mathcal A}$ that is $(\{e_1, \ldots , e_k\}, \frac{1}{k})$-Følner (and contains $\{e_1, \ldots , e_k\}$ in the case of proper algebraic amenability). We define ${\mathcal B}_{i+1}$ to be the subalgebra generated by the countably dimensional linear subspace ${\mathcal B}_i + W_1 + W_2 + \ldots$.

Now define the countably dimensional subalgebra ${\mathcal B}= \bigcup _{i=0}^\infty {\mathcal B}_i$. It is routine to verify that ${\mathcal B}$ is (properly) algebraically amenable.

Conversely, in order to check (proper) algebraic amenability of ${\mathcal A}$, we fix $\varepsilon >0$ and an arbitrary finite subset ${\mathcal F}\subset {\mathcal A}$. By assumption, ${\mathcal F}$ is contained in a countably dimensional subalgebra that is (properly) algebraically amenable, which is enough to produce the desired $({\mathcal F}, \varepsilon )$-Følner subspace. $\square$

Just as in the case of metric spaces in Sect. 2, we are interested in the distinctions and relations between amenability and proper amenability. For example, when ${\mathcal A}$ is finite dimensional, then the two notions clearly coincide. The general situation bears strong similarity to the case of metric spaces. To begin with, we present a few more ways to characterize proper algebraic amenability (for infinite dimensional algebras). The first half of the following proposition should be considered as the algebraic counterpart of what we already showed in Lemma 2.6 in the context of metric spaces.

### Proposition 3.5

Let ${\mathcal A}$ be an infinite dimensional $\mathbb {K}$-algebra. Then the following conditions are equivalent:

1. ${\mathcal A}$ is properly algebraically amenable.
2. For any $\varepsilon >0$, $N \in \mathbb {N}$ and any finite set $\mathcal {F}\subset {\mathcal A}$ there exists an $(\mathcal {F}, \varepsilon )$-Følner subspace *W* such that
  $$\begin{aligned} \dim (W) \ge N. \end{aligned}$$

When ${\mathcal A}$ is unital, they are also equivalent to

(3) For any $\varepsilon >0$ and any finite set $\mathcal {F}\subset {\mathcal A}$ there exists an $(\mathcal {F}, \varepsilon )$-Følner subspace that contains $\mathbb {1}_{{\mathcal A}}$.

### Proof

The implication (1) $\Rightarrow$ (2) is immediate from the definition, since ${\mathcal F}\subset W$ implies $\dim (W) \ge \dim ( \mathrm {span}(\mathcal {F}))$, while the latter may be made arbitrarily large since ${\mathcal A}$ is infinite dimensional.

Next we show the converse: (2) $\Rightarrow$ (1). Given any $\varepsilon >0$ and any finite set $\mathcal {F}\subset {\mathcal A}$, we may obtain from (2) a finite-dimensional linear subspace $V\subset {\mathcal A}$ such that $\dim (V) \ge \frac{4 |\mathcal {F}|}{\varepsilon }$ and

$$\begin{aligned} \frac{\dim (a V +V)}{\dim (V)}\le 1+ \frac{\varepsilon }{2}, \quad \mathrm {for~all}\quad a\in \mathcal {F}. \end{aligned}$$

Define $W := V + \mathrm {span}(\mathcal {F})$, a finite-dimensional linear subspace that contains $\mathcal {F}$. Moreover, for all $a\in \mathcal {F}$,

$$\begin{aligned} \frac{\dim (a W +W)}{\dim (W)} \le \frac{\dim (a V +V) + \dim ( \mathrm {span}(a \mathcal {F} \cup \mathcal {F}))}{\dim (V)} \le 1+ \frac{\varepsilon }{2} + \frac{\varepsilon }{2} \le 1+\varepsilon . \end{aligned}$$

This proves (1) by definition.

Now assume ${\mathcal A}$ is unital. The implication (1) $\Rightarrow$ (3) is trivial from the definition, while (3) $\Rightarrow$ (2) is also easy in view of Remark 3.2, after observing that $\mathbb {1}_{{\mathcal A}} \in W$ implies $\dim ( \mathrm {span}( \mathcal {F} W +W)) \ge \dim ( \mathrm {span}(\mathcal {F}))$. This shows that (3) is equivalent to (1) and (2). $\square$

A notable difference between algebraic amenability and proper algebraic amenability lies in their behaviors under unitization. Recall that for a (possibly unital) $\mathbb {K}$-algebra, the *unitization* of ${\mathcal A}$, denoted by $\widetilde{{\mathcal A}}$, is defined to be the unital algebra linearly isomorphic to ${\mathcal A}\oplus \mathbb {K}$, with the product defined by $(a, \lambda ) (b, \mu ) = (ab + \mu a + \lambda b, \lambda \mu )$ for any $(a, \lambda ), (b, \mu ) \in {\mathcal A}\oplus \mathbb {K}$. The element (0, 1) now serves as the unit $\mathbb {1}_{\widetilde{{\mathcal A}}}$. Observe that when ${\mathcal A}$ already has a unit, then $\widetilde{{\mathcal A}} \cong {\mathcal A}\times \mathbb {K}$ as an algebra.

### Proposition 3.6

Let ${\mathcal A}$ be a $\mathbb {K}$-algebra. Then

1. $\widetilde{{\mathcal A}}$ is algebraically amenable if ${{\mathcal A}}$ is algebraically amenable.
2. $\widetilde{{\mathcal A}}$ is properly algebraically amenable if and only if ${\mathcal A}$ is properly algebraically amenable.

### Proof

Let $\pi {:}\, {\mathcal A}\oplus \mathbb {K} \rightarrow {\mathcal A}$ be the projection onto the first coordinate and $\iota :{\mathcal A}\rightarrow {\mathcal A}\oplus \mathbb {K}$ be the embedding onto ${\mathcal A}\times \{0\}$. We also assume that ${\mathcal A}$ is infinite dimensional, as otherwise there is nothing to prove.

To prove (1), we assume ${\mathcal A}$ is algebraically amenable. Then for any $\varepsilon > 0$ and any finite subset ${\mathcal F}\subset \widetilde{{\mathcal A}}$, we pick an $(\pi ({\mathcal F}), \varepsilon )$-Følner subspace *W* in ${\mathcal A}$. Then $\iota (W) \subset \widetilde{{\mathcal A}}$ is $({\mathcal F}, \varepsilon )$-Følner because for any $(a,\lambda ) \in {\mathcal F}$, $(a,\lambda ) \cdot \iota (W) + \iota (W) = \iota (aW + W)$. Thus $\widetilde{{\mathcal A}}$ is algebraically amenable.

As for (2), we first observe that the “if” part is proved similarly as above, except for that we also use the fact that $\dim (\iota (W)) = \dim (W)$ and apply Proposition 3.5.

Conversely, suppose $\widetilde{{\mathcal A}}$ is properly algebraically amenable. For any $\varepsilon > 0$ and any finite subset ${\mathcal F}' \subset {\mathcal A}$, we pick an $(\iota ({\mathcal F}'), \varepsilon )$-Følner subspace $W'$ in $\widetilde{{\mathcal A}}$ such that $\iota ({\mathcal F}') \subset W'$. Then for any $a \in {\mathcal F}'$ and $(b, \mu ) \in W'$, we have $\iota (a) \cdot (b, \mu ) = \iota (ab + \mu a) \in \iota (ab) + W'$, and thus

$$\begin{aligned} \pi \big ( \iota (a) \cdot W' + W' \big ) = a \cdot \pi (W') + \pi (W'). \end{aligned}$$

Since $\mathrm {Ker}(\pi ) = \mathbb {K} \cdot (0,1)$, we have

$$\begin{aligned}&\frac{\dim _{\mathbb {K}}(a \cdot \pi (W') + \pi (W'))}{\dim _{\mathbb {K}}(\pi (W'))} = \frac{\dim _{\mathbb {K}}\left( \pi \big ( \iota (a) \cdot W' + W' \big )\right) }{\dim _{\mathbb {K}}(\pi (W'))} \\&\quad \in \left\{ \frac{\dim _{\mathbb {K}}\left( \iota (a) \cdot W' + W' \right) }{\dim _{\mathbb {K}}(W')} , \frac{\dim _{\mathbb {K}}\left( \iota (a) \cdot W' + W' \right) - 1}{\dim _{\mathbb {K}}(W')} , \frac{\dim _{\mathbb {K}}\left( \iota (a) \cdot W' + W' \right) -1 }{\dim _{\mathbb {K}}(W') -1 } \right\} \\&\quad \subset \left[ 1 , 1 + \frac{\dim _{\mathbb {K}}\left( \iota (a) \cdot W' + W' \right) - \dim _{\mathbb {K}}(W') }{\dim _{\mathbb {K}}(W') -1 } \right] \\&\quad \subset \left[ 1 , 1 + \varepsilon \left( 1 + \frac{1}{ | {\mathcal F}' | - 1} \right) \right] . \end{aligned}$$

Since without loss of generality, we may assume $|{\mathcal F}'| \ge 2$, thus $\pi (W')$ is $({\mathcal F}', 2 \varepsilon )$-Følner and contains ${\mathcal F}'$. This shows that ${{\mathcal A}}$ is properly algebraically amenable. $\square$

The following example exhibits the difference between algebraic amenability and proper algebraic amenability, and also demonstrate that the converse of (1) in Proposition 3.6 is false (see also Theorem 3.2 in [45] for an operator theoretic counterpart).

### Example 3.7

Let ${\mathcal A}$ be a $\mathbb {K}$-algebra with a non-zero left ideal *I* of finite $\mathbb K$-dimension. Then ${\mathcal A}$ is always algebraically amenable, since *I* is an $({\mathcal A}, \varepsilon =0)$-Følner subspace. Therefore an easy way to construct an amenable $\mathbb {K}$-algebra that is not properly amenable is to take a direct sum of a finite dimensional algebra and a non-algebraically-amenable algebra (e.g., the group algebra of a non-amenable group; see Example 3.12). In particular, if ${\mathcal A}$ is a non-amenable unital algebra, then $\widetilde{{\mathcal A}} \cong {\mathcal A}\oplus \mathbb {K}$ is algebraically amenable but not properly algebraically amenable. Moreover, this is the only way in which a unitization $\widetilde{{\mathcal A}}$ can be algebraically amenable but not properly algebraically amenable, as we will show in Corollary 3.11.

The next result refers to two-sided ideals.

### Proposition 3.8

Let ${\mathcal A}$ be a $\mathbb {K}$-algebra with a non-zero two-sided ideal *I* of finite $\mathbb K$-dimension. Then, ${\mathcal A}$ is properly algebraically amenable if and only if the quotient algebra ${\mathcal A}/I$ is.

### Proof

Let $\pi :{\mathcal A}\rightarrow {\mathcal A}/ I$ is the natural projection, then for any $\varepsilon >0$ and any finite set $\mathcal {F}\subset {\mathcal A}$, $V \mapsto \pi ^{-1}(V)$ defines a map from  to  with $\mathrm {dim} (\pi ^{-1}(V) ) \ge \mathrm {dim} (V)$.

On the other hand for any $\varepsilon >0$ and any finite set $\mathcal {F'}\subset {\mathcal A}/ I$ such that $\mathrm {dim} (\mathrm {span} (\mathcal F ')) > 0$, $W \mapsto \pi (W)$ defines a map from  to  with

$$\begin{aligned} \mathrm {dim}( \pi (W)) = \mathrm {dim}(W) - \mathrm {dim}(I), \end{aligned}$$

where

$$\begin{aligned} K= 1 + \frac{\mathrm {dim} (\pi ^{-1} (\mathrm {span}(\mathcal F ')))}{\mathrm {dim} (\pi ^{-1} (\mathrm {span}(\mathcal F '))) - \mathrm {dim}(I)}, \end{aligned}$$

and  is the set of all *W* in  such that $V\subseteq W$, for any finite-dimensional subspace *V* of $\mathcal A$. Indeed, for *W* in , we have

$$\begin{aligned}&\frac{\mathrm {dim} (\pi (aW+W))}{\mathrm {dim} (\pi (W))} = \frac{\mathrm {dim} (aW+W) -\mathrm {dim} (I)}{ \text {dim} (W) - \text {dim}(I)} \\&\quad = \frac{\text {dim} (aW+W)}{\text {dim}(W)} \cdot \frac{\text {dim} (W)}{\text {dim} (W) - \text {dim} (I)} + \Big ( 1 - \frac{\text {dim} (W)}{\text {dim} (W) - \text {dim} (I)} \Big ) \\&\quad = \Big ( \frac{\text {dim} (aW+W)}{\text {dim} (W)} -1 \Big ) \Big ( \frac{\text {dim} (W)}{\text {dim} (W)- \text {dim} (I)} \Big ) +1 \\&\quad \le \frac{\varepsilon }{K} \Big ( \frac{\text {dim}(W)}{\text {dim} (W)- \text {dim} (I)} \Big ) +1 , \end{aligned}$$

and it is easily seen that

$$\begin{aligned}&\frac{1}{K}\Big ( \frac{\text {dim}(W)}{\text {dim} (W)- \text {dim} (I)} \Big ) \\&\qquad \qquad = \Big ( \frac{\mathrm {dim} (\pi ^{-1} (\mathrm {span}(\mathcal F ')))- \mathrm {dim}(I)}{ 2 \mathrm {dim} (\pi ^{-1} (\mathrm {span}(\mathcal F '))) - \mathrm {dim}(I)} \Big ) \Big ( \frac{\text {dim}(W)}{\text {dim} (W)- \text {dim} (I)}\Big ) \le 1, \end{aligned}$$

giving the result. $\square$

Next we show that the only situation where algebraic amenability and proper algebraic amenability differ is when the $\mathbb K$-algebra contains a non-zero left ideal of finite $\mathbb K$-dimension, as demonstrated by the following theorem. This situation is similar to what is known for Hilbert space operators (cf., [45, Theorem 4.1]).

### Theorem 3.9

Let ${\mathcal A}$ be an infinite dimensional $\mathbb {K}$-algebra that is algebraically amenable but not properly algebraically amenable. Then there exists a nonzero element $a \in {\mathcal A}$ with

$$\begin{aligned} \dim ({\mathcal A}\cdot a) < \infty . \end{aligned}$$

### Proof

Since the algebra ${\mathcal A}$ is fixed we will denote for simplicity the collection  of Følner $({\mathcal F},\varepsilon )$-subspaces of ${\mathcal A}$ by . Since ${\mathcal A}$ is algebraically amenable, we know that for any $\varepsilon > 0$ and any finite set $\mathcal {F} \subset {\mathcal A}$ the collection . Hence we may defineOn the other hand, as ${\mathcal A}$ is not properly algebraically amenable, by condition (2) of Proposition 3.5, there exist $\varepsilon _0 > 0$ and finite set $\mathcal {F}_0 \subset {\mathcal A}$ such that $N_{\mathcal {F}_0, \varepsilon _0} < \infty$. Since $N_{\mathcal {F}, \varepsilon }$ is increasing with respect to $\varepsilon$, without loss of generality we may assume that $\varepsilon _0 \cdot N_{\mathcal {F}_0, \varepsilon _0} < 1$.

For any $\varepsilon \in (0, \varepsilon _0]$ and finite set $\mathcal {F} \subset {\mathcal A}$ containing $\mathcal {F}_0$, we claim thatIndeed, the inclusion $\supseteq$ is clear. On the other hand, for any  and $a \in \mathcal {F}$, we have

$$\begin{aligned} \dim (a W + W)\le & {} (1 + \varepsilon ) \dim (W) \;\le \; \dim (W) + \varepsilon N_{\mathcal {F}, \varepsilon } \\\le & {} \dim (W) + \varepsilon _0 \cdot N_{\mathcal {F}_0, \varepsilon _0} \;<\; \dim (W) + 1. \end{aligned}$$

Since $\dim (a W + W) \ge \dim (W)$ and from the fact that dimensions are in $\mathbb {N}_0$ we conclude that $\dim (aW + W) = \dim (W)$.

Observe that a non-zero finite-dimensional linear subspace *W* of ${\mathcal A}$ is $(\mathcal {F}, 0)$-Følner iff $\mathcal {F} \cdot W \subset W$. For any finite set $\mathcal {F} \subset {\mathcal A}$ containing $\mathcal {F}_0$, since by what we have shown,  is a non-empty finite subset of $\mathbb {N}$, we haveis not empty. Furthermore for any finite set $\mathcal {F}' \subset {\mathcal A}$ containing $\mathcal {F}$, and for any  and , we claim that $W' \subseteq W$. Indeed, if this were not the case, then $W + W'$ would be a member of  with dimension strictly greater than $\dim (W)$, contradicting the definition of . Notice that by setting $\mathcal {F}' = \mathcal {F}$, this claim implies that  contains only one element, which we now denote as $W_\mathcal {F}$.

Consider the decreasing net $\{ \dim (W_\mathcal {F}) \}_{\mathcal {F} \in \mathcal {J}}$ indexed by

$$\begin{aligned} \mathcal {J} := \{\mathcal {F} \subset {\mathcal A}\ : |\mathcal {F}| < \infty , \ \mathcal {F}_0 \subset \mathcal {F} \}. \end{aligned}$$

Since its range is contained in the finite set $\mathbb {Z}\cap [1, \dim (W_{\mathcal {F}_0})]$, we see that $\displaystyle \lim \nolimits _{\mathcal {F} \in \mathcal {J}} \dim (W_\mathcal {F})$ exists and is realized by some member $W_{\mathcal {F}_1}$. It follows that $W_\mathcal {F} = W_{\mathcal {F}_1}$ for any finite $\mathcal {F} \subset {\mathcal A}$ containing $\mathcal {F}_1$, and thus $a \cdot W_{\mathcal {F}_1} \subseteq W_{\mathcal {F}_1}$ for any $a \in {\mathcal A}$, i.e., $W_{\mathcal {F}_1}$ is a non-zero left ideal with finite $\mathbb {K}$-dimension. Consequently, if we pick any $a \in W_{\mathcal {F}_1}$, then

$$\begin{aligned} \dim ({\mathcal A}\cdot a) \le \dim (W_{\mathcal {F}_1}) < \infty \end{aligned}$$

and the proof is concluded. $\square$

### Corollary 3.10

Let ${\mathcal A}$ be a $\mathbb K$-algebra without zero-divisor, then ${\mathcal A}$ is algebraically amenable if and only if it is properly algebraically amenable.

### Proof

We only need to prove the case when ${\mathcal A}$ is infinite-dimensional. Since ${\mathcal A}$ has no zero-divisor, for any non-zero $a \in {\mathcal A}$ and finite subset ${\mathcal F}\subset {\mathcal A}$, we have

$$\begin{aligned} \dim (\mathrm {span}({\mathcal F}) \, a) = \dim (\mathrm {span}({\mathcal F})). \end{aligned}$$

This clearly contradicts the conclusion of Theorem 3.9, and thus its hypothesis cannot hold. $\square$

### Corollary 3.11

Suppose that ${\mathcal A}$ is a non-algebraically amenable algebra such that its unitization $\widetilde{{\mathcal A}}$ is algebraically amenable. Then ${\mathcal A}$ is a unital algebra.

### Proof

By Proposition 3.6 (2), $\widetilde{{\mathcal A}}$ is not properly algebraically amenable and so, by Theorem 3.9, $\widetilde{{\mathcal A}}$ contains a nonzero finite-dimensional left ideal *I*. Since ${\mathcal A}$ is not algebraically amenable, we must have $I\cap {\mathcal A}= \{0\}$, and it follows that *I* is one-dimensional and that $I\oplus {\mathcal A}= \widetilde{{\mathcal A}}$. Let $(b,1)\in \widetilde{{\mathcal A}}$, where $b\in {\mathcal A}$. Then $(a,0)(b,1) \in I$ implies that $a(-b)= a$ for all $a\in {\mathcal A}$, so that $e:= -b$ is a right unit for ${\mathcal A}$. In particular, *e* is idempotent and ${\mathcal A}= {\mathcal A}e$. If

$$\begin{aligned} (1-e) {\mathcal A}= \{ a-ea : a\in {\mathcal A}\} \end{aligned}$$

is nonzero, then any nonzero finite-dimensional linear subspace of $(1-e) {\mathcal A}$ is an $(\mathcal F , 0)$-Følner subspace for every finite subset $\mathcal F$ of ${\mathcal A}$, and so ${\mathcal A}$ is algebraically amenable, contradicting our assumption. Therefore $(1-e){\mathcal A}= 0$ and ${\mathcal A}$ is unital with unit *e*.$\square$

### Example 3.12

([15, Corollary 4.5]) The group algebra $\mathbb {K}G$ is algebraically amenable if and only if it is properly algebraically amenable if and only if *G* is amenable.

## Paradoxical decompositions and invariant dimension measures of $\mathbb {K}$-algebras

Elek showed that, analogous to the situation for groups, there is a dichotomy between algebraic amenability and a certain kind of paradoxical decomposition defined for algebras (cf., [32, Theorem 2]). However, in his paper, the conditions of countable dimensionality and the non-existence of zero-divisors are required.

We remark here that these conditions can be removed if one replaces Elek’s definition [corresponding to *proper* algebraic amenability as in Definition 3.1 (ii)] with algebraic amenability as in Definition 3.1 (i). By Theorem 3.9 the assumption of no zero-divisors happens to have the effect that the properness for algebraic amenability comes for free. We will state and prove this general version of Elek’s theorem below.

We recall some definitions, adapted to our needs. When working with a zero-divisor *r*, it is useful to restrict attention to subspaces *A* where *r* acts non-degenerately. More precisely, if *A* is a linear subspace of $\mathcal A$, we say that $r|_A$ is injective if the map $a\mapsto ra$ given by left multiplication by *r* is injective on *A*. Equivalently, $A\cap \mathrm {r.ann}(r) = \{ 0 \}$, where

$$\begin{aligned} \mathrm {r.ann} (r) = \{ x\in \mathcal A : rx=0 \} \end{aligned}$$

is the right annihilator of *r*.

The following definition of paradoxicality is equivalent to the one given by Elek in [32]. We prefer this formulation because it is formally closer to the usual condition for actions of groups, (cf., [54, Definition 1.1]).

### Definition 4.1

Let ${\mathcal A}$ be a $\mathbb K$-algebra. Let $\{ e_i \}_{i \in I}$ be a basis of ${\mathcal A}$ over $\mathbb K$ and $\mathcal {S}$ a subset of ${\mathcal A}$. A *paradoxical decomposition* of $\{ e_i \}_{i \in I}$ by $\mathcal {S}$ consists of two partitions $(L_0, L_1, \ldots , L_n)$ and $(R_0, R_1, \ldots , R_m)$ of $\{ e_i \}_{i \in I}$, i.e.

$$\begin{aligned} \{ e_i \}_{i \in I} = L_0\sqcup L_1\sqcup \ldots \sqcup L_n = R_0\sqcup R_1 \sqcup \ldots \sqcup R_m, \end{aligned}$$

together with elements $g_1,\ldots ,g_n, h_1,\ldots , h_m \in \mathcal {S}$, such that

$$\begin{aligned} L_0 \cup g_1L_1 \cup \ldots \cup g_nL_n \cup R_0 \cup h_1R_1 \cup \ldots \cup h_mR_m \end{aligned}$$

is a disjoint union and linearly independent family in ${\mathcal A}$.

If such a paradoxical decomposition exists, we say $\{ e_i \}_{i \in I}$ is *paradoxically decomposed* by $\mathcal {S}$.

Note that, in particular, $g_i|_{A_i}$ and $h_j|_{B_j}$ are injective, where $A_i$ is the linear span of $L_i$ and $B_j$ is the linear span of $R_j$.

### Remark 4.2

(i) The slight formal inhomogeneity with $L_0$ and $R_0$ can be fixed by adding the unit $\mathbb {1}_{\mathcal A}$ into $\mathcal {S}$, when ${\mathcal A}$ is unital. This way, we may write $L_0$ as $\mathbb {1}_{\mathcal A}L_0$, and $R_0$ as $\mathbb {1}_{\mathcal A}R_0$. When ${\mathcal A}$ is not unital, we can still fix it by considering $\mathcal {S}$ as a subset of $\widetilde{{\mathcal A}}$ and adding $\mathbb {1}_{\widetilde{{\mathcal A}}}$ into it.
(ii) Following [32, Definition 1.2], we may also present a variant of the above definition involving only one partition. Namely, we define a *one-partition paradoxical decomposition* of $\{ e_i \}_{i \in I}$ by $\mathcal {S}$ so that it consists of a partition $\{ e_i \} _{i \in I} = T_1 \sqcup \ldots \sqcup T_k$ and elements $g_1,\ldots ,g_k, h_1,\ldots , h_k \in \mathcal {S}$ with the property that
  $$\begin{aligned} g_1T_1 \cup \ldots \cup g_k T_k \cup h_1T_1 \cup \ldots \cup h_kT_k \end{aligned}$$
  is a disjoint union and linearly independent family in ${\mathcal A}$. Though this is seemingly a more restrictive notion, the existence of this one-partition version is equivalent to that of a general paradoxical decomposition, provided that $\mathcal {S}$ contains the unit (of ${\mathcal A}$ or $\widetilde{{\mathcal A}}$). Indeed, starting from a general paradoxical decomposition
  $$\begin{aligned} \big ((L_0, \ldots , L_n), (R_0, \ldots , R_m), (g_1,\ldots ,g_n), (h_1,\ldots , h_m) \big ), \end{aligned}$$
  we may define a one-partition paradoxical decomposition by setting $T_{ij}: = L_i\cap R_j$, $g_{ij}:= g_i$, and $h_{ij}:= h_j$ for $i=0,\ldots , n$ and $j=0,\ldots , m$, with the understanding that $g_0 = h_0 = \mathbb {1}_{\mathcal A}$ or $\mathbb {1}_{\widetilde{{\mathcal A}}}$.
(iii) The relation to Elek’s definition in [32] is thus as follows: a unital countably dimensional algebra is *paradoxical* in the sense of [32, Definition 1.2] if and only if for any (countable) basis $\{ e_i \}_{i \in I}$ of ${\mathcal A}$, there is a paradoxical decomposition of $\{ e_i \}_{i \in I}$ by ${\mathcal A}$.

The following lemma generalizes [32, Lemma 2.2].

### Lemma 4.3

Fix $\lambda > 1$. Then a $\mathbb K$-algebra ${\mathcal A}$ is not algebraically amenable if and only if there exists a finite subset ${\mathcal F}\subset {\mathcal A}$, such that for any nonzero finite dimensional linear subspace $W \subset {\mathcal A}$, we have

$$\begin{aligned} \frac{\dim ({\mathcal F}W +W)}{\dim (W)} > \lambda . \end{aligned}$$

### Proof

By inverting the condition in Remark 3.2, we see that ${\mathcal A}$ is not algebraically amenable if and only if there exists $\varepsilon > 0$ and finite subset ${\mathcal F}\subset {\mathcal A}$, such that for any nonzero finite dimensional linear subspace $W \subset {\mathcal A}$, we have

$$\begin{aligned} \frac{\dim ({\mathcal F}W +W)}{\dim (W)} > 1+\varepsilon . \end{aligned}$$

This proves the “if” part. For the “only if” part, we observe that $\varepsilon$ can be taken to be arbitrarily large: we set

$$\begin{aligned} {\mathcal F}^{(n)} = \big \{a_1 \cdots a_m \ | \ m \in \{1, \ldots , n\}, \ \ a_k \in {\mathcal F}_0, \ \forall k \in \{1, \ldots , m\} \big \}. \end{aligned}$$

Then by induction we have

$$\begin{aligned} \frac{\dim ({\mathcal F}_0^{(n)} W +W)}{\dim (W)} > (1+\varepsilon )^n. \end{aligned}$$

For our purpose, we fix ${\mathcal F}' = {\mathcal F}^{(\lceil \log _{1+\varepsilon } \lambda \rceil +1)}$, so that

$$\begin{aligned} \frac{\dim ({\mathcal F}' W +W)}{\dim (W)} > \lambda . \end{aligned}$$

Replacing ${\mathcal F}$ by ${\mathcal F}'$ proves the “only if” direction. $\square$

The following is a key proposition of this section. It generalizes Proposition 2.2 in [32] to arbitrary $\mathbb K$-algebras which may have zero-divisors, have no unit, or have uncountable dimensions. To prove this, we adapt ideas from [40, Theorem 3.4, (vi) $\Rightarrow$ (v)] (see also [40]) in the context of groups and metric spaces to the algebraic setting.

### Proposition 4.4

Assume that ${\mathcal A}$ is a $\mathbb K$-algebra which is not algebraically amenable. Then there exists a finite subset ${\mathcal F}\subset {\mathcal A}$ such that for any basis $\{e_i\}_{i \in I}$ of ${\mathcal A}$, there is a paradoxical decomposition of $\{e_i\}_{i \in I}$ by ${\mathcal F}$.

### Proof

By Lemma 4.3, there exists a finite subset ${\mathcal F}\subset {\mathcal A}$, such that for any nonzero finite dimensional linear subspace $W \subset {\mathcal A}$, we have

$$\begin{aligned} \frac{\dim ({\mathcal F}W +W)}{\dim (W)} > 2. \end{aligned}$$

Such a local doubling behavior of ${\mathcal F}$ can be seen as a local form of paradoxicality, which we will now exploit to produce a paradoxical decomposition for any basis $\{e_i\}_{i \in I}$ of ${\mathcal A}$. To this end, we define ${\mathcal F}^+ = {\mathcal F}\sqcup \{*\}$, where $*$ is an abstract element, for which we prescribe a multiplication $* \cdot e_i = e_i$ for any $i \in I$ (thus $*$ behaves like a unit). Define $\Omega$ to be the set of maps $\omega :I \times \{0,1\} \rightarrow \mathcal {P}({\mathcal F}^+)$ (the power set of ${\mathcal F}^+$) with the property that for any finite subset $K \subset I \times \{0,1\}$,

$$\begin{aligned} \dim _{\mathbb K} \Bigg ( \mathrm {span}_{\mathbb K} \bigg ( \bigcup _{(i, j) \in K} \bigcup _{a \in \omega (i,j)} a \cdot e_i \bigg ) \Bigg ) \ge | K |. \end{aligned}$$

Notice that $\Omega$ is nonempty: the constant function with value ${\mathcal F}^+$ lives in $\Omega$ because of the local doubling behavior of ${\mathcal F}$.

Our goal is to “trim down” the above constant set-valued function to a singleton-valued function in $\Omega$. For this purpose, we use the natural partial order on $\Omega$ given by pointwise inclusion: $\omega \le \omega '$ if $\omega (i,j) \subset \omega '(i,j)$ for any $(i,j) \in I \times \{0,1\}$. Since any descending chain in $\Omega$ has a non-empty lower bound given by pointwise intersection, by Zorn’s Lemma, we can find a minimal element $\omega _0 \in \Omega$.

We claim that $| \omega _0(i,j) | = 1$ for any $(i,j) \in I \times \{0,1\}$. Firstly, since

$$\begin{aligned} \dim _{\mathbb K} \bigg ( \mathrm {span}_{\mathbb K} \Big ( \bigcup _{a \in \omega _0(i,j)} a \cdot e_i \Big ) \bigg ) \ge |\{(i,j)\} | = 1 \; , \end{aligned}$$

we only need to show $| \omega _0(i,j) | \le 1$. Then, suppose this were not the case: then there exists an index $(i,j)\in I\times \{0,1\}$ and two distinct elements $a_0, a_1 \in \omega _0(i,j)$. Notice that the minimality of $\omega _0$ implies that for $l \in \{0,1\}$, we can find a finite subset $K_l \subset I \times \{0,1\}$ not containing (*i*, *j*), such that

$$\begin{aligned} \dim _{\mathbb K} \Bigg ( \mathrm {span}_{\mathbb K} \bigg ( \Big ( \bigcup _{(i', j') \in K_l} \bigcup _{a \in \omega _0(i',j')} a \cdot e_{i'} \Big ) \cup \Big ( \bigcup _{a \in \omega _0(i,j) {\setminus } \{a_l\} } a \cdot e_i \Big ) \bigg ) \Bigg ) \le |K_l|, \end{aligned}$$

since otherwise if no such $K_l$ exists, we would be able to remove $a_l$ from $\omega _0(i,j)$ to produce a new element in $\Omega$ strictly smaller than $\omega _0$.

Now because of the simple fact that $(\omega _0(i,j){{\setminus }}\{a_0\} ) \cup (\omega _0(i,j){\setminus }\{a_1\} ) = \omega _0(i,j)$, we would see that, if we denote

$$\begin{aligned} W_l := \mathrm {span}_{\mathbb K} \bigg ( \Big ( \bigcup _{(i', j') \in K_l} \bigcup _{a \in \omega _0(i',j')} a \cdot e_{i'} \Big ) \cup \Big ( \bigcup _{a \in \omega _0(i,j) {\setminus } \{a_l\} } a \cdot e_i \Big ) \bigg ) \end{aligned}$$

for $$l \in \{0,1\}$$, then

$$\begin{aligned} | K_0| + | K_1 |\ge & {} \dim _{\mathbb K} (W_0) + \dim _{\mathbb K} (W_1) \\= & {} \dim _{\mathbb K} (W_0 + W_1) + \dim _{\mathbb K} (W_0 \cap W_1) \\\ge & {} \dim _{\mathbb K} \Bigg ( \mathrm {span}_{\mathbb K} \bigg ( \Big ( \bigcup _{(i', j') \in K_0 \cup K_1} \bigcup _{a \in \omega _0(i',j')} a \cdot e_{i'} \Big ) \cup \Big ( \bigcup _{a \in \omega _0(i,j) } a \cdot e_i \Big ) \bigg ) \Bigg ) \\&+ \dim _{\mathbb K} \Bigg ( \mathrm {span}_{\mathbb K} \Big ( \bigcup _{(i', j') \in K_0 \cap K_1} \bigcup _{a \in \omega _0(i',j')} a \cdot e_{i'} \Big ) \Bigg ) \\\ge & {} |K_0 \cup K_1 \cup \{(i,j)\}| + |K_0 \cap K_1| \\= & {} |K_0 \cup K_1| + 1 + |K_0 \cap K_1| \\= & {} |K_0| + |K_1| + 1, \end{aligned}$$

which gives a contradiction. Hence we have proved our claim that $| \omega _0(i,j)| = 1$ for any $(i,j) \in I \times \{0,1\}$.

Thus we may define $\phi :I \times \{0,1\} \rightarrow {\mathcal F}^+$ such that $\omega _0(i,j) = \{ \phi (i,j) \}$. It follows from the defining property of $\Omega$ that $\phi$ satisfies

$$\begin{aligned} \dim _{\mathbb K} \bigg ( \mathrm {span}_{\mathbb K} \Big ( \bigcup _{(i, j) \in K} \phi (i,j) \cdot e_i \Big ) \bigg ) = | K | \end{aligned}$$

for any finite subset $K \subset I \times \{0,1\}$, i.e., $\{ \phi (i,j) \cdot e_i \}_{(i,j) \in I \times \{0,1\} }$ is a linearly independent family in ${\mathcal A}$.

To conclude the proof, we define, for each $a \in {\mathcal F}^+$,

$$\begin{aligned} L_a =&\ \{ e_i \ | \ i \in I, \phi (i,0) = a \} \\ R_a =&\ \{ e_i \ | \ i \in I, \phi (i,1) = a \}. \end{aligned}$$

Therefore we have two finite partitions

$$\begin{aligned} \{e_i\}_{i\in I} = L_* \sqcup \bigsqcup _{a \in {\mathcal F}} L_a = R_* \sqcup \bigsqcup _{a \in {\mathcal F}} R_a \end{aligned}$$

such that

$$\begin{aligned} \Big ( L_* \cup \bigcup _{a \in {\mathcal F}} a L_a \Big ) \cup \Big ( R_* \cup \bigcup _{a \in {\mathcal F}} a R_a \Big ) \end{aligned}$$

is a disjoint union and linearly independent family in ${\mathcal A}$. Thus we have produced a paradoxical decomposition of $\{e_i\}_{i\in I}$ by ${\mathcal F}$ in the sense of Definition 4.1. $\square$

Now we define a suitable notion of invariant dimension-measure for $\mathbb {K}$-algebras, an analogue of invariant mean for amenable groups. Note that the lack of distributivity in the lattice of subspaces of a vector space makes it necessary to give up some of the properties one would expect for this concept.

### Definition 4.5

Let ${\mathcal A}$ be a $\mathbb K$-algebra and $\{e_i \}_{i \in I}$ be a $\mathbb K$-linear basis of ${\mathcal A}$. A *dimension-measure* on ${\mathcal A}$ associated to $\{e_i \}_{i \in I}$ is a function $\mu$ from the set of linear subspaces of ${\mathcal A}$ to [0, 1] which satisfies the following properties:

(i) $$ \mu ({\mathcal A}) =1$$.
(ii) If *A*, *B* are linear subspaces in ${\mathcal A}$ with $A\,\cap \, B = \{0 \}$, then $\mu (A\,\oplus \, B) \ge \mu (A)\,+\, \mu (B)$.
(iii) For every partition $L_1\sqcup L_2\sqcup \ldots \sqcup L_m$ of $\{e_i \}_{i \in I}$, we have $\sum _{k=1}^m \mu (\mathrm {span}(L_k)) =1$.

Let $\mathcal {S}$ be a subset of ${\mathcal A}$. We say $\mu$ is $\mathcal {S}$ *-invariant* if

(iv) For any $s\in \mathcal {S}$ and any linear subspace $A \subset {\mathcal A}$ such that $s|_A$ is injective, we have $\mu (sA) \ge \mu (A)$.

Note that if $\mu$ is a dimension-measure on ${\mathcal A}$ and $A\subseteq B$ are subspaces of ${\mathcal A}$, then, by property (ii), it follows that $\mu (A) \le \mu (B)$.

We can now state the following generalization of [32, Theorem 1].

### Theorem 4.6

Let ${\mathcal A}$ be a $\mathbb {K}$-algebra. Then the following conditions are equivalent:

1. ${\mathcal A}$ is algebraically amenable.
2. For any finite subset ${\mathcal F}\subset {\mathcal A}$, there is a basis of ${\mathcal A}$ that cannot be paradoxically decomposed by ${\mathcal F}$.
3. For any countably dimensional linear subspace $W \subset {\mathcal A}$, there is a basis of ${\mathcal A}$ that cannot be paradoxically decomposed by *W*.
4. For any countably dimensional linear subspace $W \subset {\mathcal A}$, there exists a *W*-invariant dimension-measure on ${\mathcal A}$ (associated to some basis).

### Proof

The implication (2) $\Rightarrow$ (1) follows from Proposition 4.4. The implication (3) $\Rightarrow$ (2) is immediate by setting $W = \mathrm {span}({\mathcal F})$.

To show (4) $\Rightarrow$ (3), we fix an arbitrary countably dimensional linear subspace $W \subset {\mathcal A}$. By (4), there is a basis $\{ e_i \}_{i \in I}$ of ${\mathcal A}$ and a *W*-invariant dimension-measure $\mu$ on ${\mathcal A}$ associated to $\{ e_i \}_{i \in I}$. Suppose there were a paradoxical decomposition

$$\begin{aligned} \big ( (L_0, \ldots , L_n), (R_0, \ldots , R_m), (g_1, \ldots , g_n), (h_1, \ldots , h_m) \big ) \end{aligned}$$

of $\{ e_i \}_{i \in I}$ by *W*. Put $A_k := \text {span}(L_k)$ and $B_l := \text {span}(R_l)$. We have $\sum _{k=0}^n \mu (A_k) = 1 = \sum _{l=0}^m \mu (B_l)$ (by (iii) in Definition 4.5). Also $g_k|_{A_k}$ and $h_l|_{B_l}$ are injective for all *k*, *l* and so $\mu (g_kA_k )\ge \mu (A_k)$ and $\mu (h_lB_l) \ge \mu (B_l)$ for all *k*, *l* (by (iii)), so that we get

$$\begin{aligned} 1&\ge \mu (A_0 \oplus g_1A_1\oplus \ldots g_nA_n\oplus B_0 \oplus h_1B_1\oplus \ldots \oplus h_mB_m)\\&\ge \mu (A_0) + \sum _{k=1}^n \mu (g_kA_k) + \mu (B_0) + \sum _{l=1}^m \mu (h_lB_l)\\&\ge \sum _{k=0}^n \mu (A_k) + \sum _{l=0}^m \mu (B_l) = 2, \end{aligned}$$

which is a contradiction.

Finally, to show (1) $\Rightarrow$ (4) we construct, for an arbitrary countably dimensional linear subspace $W \subset {\mathcal A}$, a dimension-measure $\mu$ on ${\mathcal A}$ associated to some basis. This involves two cases:

1. ${\mathcal A}$ is properly algebraically amenable. By Proposition 3.4, there is a countably dimensional subalgebra ${\mathcal B}\subset {\mathcal A}$ that is properly algebraically amenable and contains *W*. Let $\{ W_i \}_{i=1}^{\infty }$ be an increasing sequence of finite-dimensional subspaces of ${\mathcal A}$ such that ${\mathcal B}= \cup _{i=1}^{\infty } W_i$, and such that
  $$\begin{aligned} \lim _{i\rightarrow \infty } \frac{\dim (aW_i+W_i)}{\dim (W_i)} = 1 \end{aligned}$$
  for all $a\in {\mathcal B}$. Let $\omega$ be a free ultrafilter on $\mathbb N$, and let $\{e_i \}_{i=1}^{\infty }$ be a basis for ${\mathcal B}$ obtained by successively enlarging basis of the spaces $W_i$ (cf. [32, Proposition 2.1]). We then enlarge $\{e_i \}_{i=1}^{\infty }$ to a basis $\{e_i \}_{i \in I}$ of ${\mathcal A}$, where $\mathbb {N} \subset I$. For a linear subspace *A* of ${\mathcal A}$, set
  $$\begin{aligned} \mu (A) = \lim _{\omega } \frac{\dim (A\cap W_i)}{\dim (W_i)}. \end{aligned}$$
  Obviously, we have $\mu ({\mathcal A}) =1$ and $0 \le \mu (A)\le 1$ for every subspace *A*. Moreover, properties (ii) and (iii) in Definition 4.5 clearly hold, so we only need to check (iv). To prove (iv) we first show that for any $a\in W$ and any linear subspace *A* we have
  4.1 $$\begin{aligned} \mu (A) = \lim _{\omega }\frac{\dim ( (W_i+aW_i)\cap A ) }{\dim (W_i)}. \end{aligned}$$
  Write $T_i = (W_i+aW_i)\cap A$. Then $T_i\cap W_i = A\cap W_i$, so that $T_i = (W_i\cap A)\oplus T_i'$ with $T_i'\cap W_i = \{ 0\}$. Hence
  $$\begin{aligned} \frac{\dim (T_i)}{\dim (W_i)} = \frac{\dim (W_i\cap A)}{\dim (W_i)} + \frac{\dim (T_i')}{\dim (W_i)}. \end{aligned}$$
  Since $\dim (T_i') /\dim (W_i)\rightarrow 0$, we obtain the result. We now show (iv). Let $a \in W$ be such that $a|_A$ is injective. Then we have
  $$\begin{aligned} \mu (aA)&= \lim _{\omega } \frac{\dim ((W_i+aW_i)\cap aA)}{\dim (W_i)} \ge \lim _{\omega } \frac{\dim (aW_i\cap aA)}{\dim (W_i)}\\&\ge \lim _{\omega } \frac{\dim (a(W_i\cap A))}{\dim (W_i)} = \lim _{\omega } \frac{\dim (W_i \cap A)}{\dim (W_i)}= \mu (A) , \end{aligned}$$
  where in the second equality we have used that *a*|*A* is injective.
2. ${\mathcal A}$ is algebraically amenable but not properly algebraically amenable. By Theorem 3.9, we only need to build a dimension-measure in the case where ${\mathcal A}$ has a nonzero finite-dimensional left ideal *I*. This is easily taken care of by defining
  $$\begin{aligned} \mu (A) = \frac{\dim (I\cap A)}{\dim (I)} \end{aligned}$$
  for each linear subspace $A \subset {\mathcal A}$.

This concludes the proof of Theorem. $\square$

For countably dimensional (or equivalently, countably generated) $\mathbb {K}$-algebras, the statement of the previous theorem can be somewhat simplified:

### Corollary 4.7

Let ${\mathcal A}$ be a countably dimensional $\mathbb {K}$-algebra. Then the following conditions are equivalent:

1. ${\mathcal A}$ is algebraically amenable.
2. There is a basis of ${\mathcal A}$ that cannot be paradoxically decomposed by ${\mathcal A}$.
3. There exists an ${\mathcal A}$-invariant dimension-measure on ${\mathcal A}$ (associated to some basis).

### Proof

This is immediate after we set $W = {\mathcal A}$ in the statement of Theorem 4.6.$\square$

### Remark 4.8

If $\mu$ is as build before, and *a* is a non-zero-divisor in ${\mathcal A}$, then one gets $\mu (aA)= \mu (A)$ (cf., [32]). The reason is that, in this case, we have

$$\begin{aligned} \dim (a^{-1}W_i + W_i ) \le \dim (W_i + aW_i), \end{aligned}$$

where $a^{-1}W_i= \{ x\in {\mathcal A}: ax\in W_i \}$, because left multiplication by *a* induces an injective map from $a^{-1}W_i+W_i$ into $W_i+aW_i$. Therefore we get

$$\begin{aligned} \lim _i \frac{\dim (a^{-1}W_i+W_i)}{\dim (W_i)} = 1. \end{aligned}$$

Hence, for any linear subspace *A* of ${\mathcal A}$, we can show

$$\begin{aligned} \mu (A) = \lim _{\omega } \frac{\dim ((a^{-1}W_i+W_i)\cap A)}{\dim (W_i)} \end{aligned}$$

just as in the proof of Eq. (4.1).

Moreover, we have

$$\begin{aligned} \frac{\dim (a(a^{-1}W_i))}{\dim (W_i)} \ge \frac{\dim (W_i \cap aW_i)}{\dim (W_i)} \rightarrow 1 \end{aligned}$$

and thus,

$$\begin{aligned} \mu (B) = \lim _{\omega } \frac{\dim (a(a^{-1}W_i) \cap B)}{\dim (W_i)} \end{aligned}$$

for any linear subspace *B* of ${\mathcal A}$. We obtain

$$\begin{aligned} \mu (A)&= \lim _{\omega } \frac{\dim ((a^{-1}W_i+W_i)\cap A)}{\dim (W_i)} \ge \lim _{\omega } \frac{\dim (a^{-1}W_i\cap A)}{\dim (W_i)}\\&= \lim _{\omega } \frac{\dim (a(a^{-1}W_i)\cap aA)}{\dim (W_i)} = \mu (aA). \end{aligned}$$

This proves our claim. $\square$

Recall the usual Murray–von Neumann equivalence $\sim$ and comparison $\gtrsim$ for idempotents of an algebra, defined as follows: for idempotents *e*, *f* in ${\mathcal A}$, write $e\sim f$ if there are $x,y\in {\mathcal A}$ such that $e=xy$ and $f=yx$; write $e \gtrsim f$ if there are $x,y\in {\mathcal A}$ such that $xy \in e{\mathcal A}e$ and $f=yx$. These relations naturally extends to the infinite matrix algebra $M_\infty ({\mathcal A}) := \bigcup _{n=1}^\infty M_n({\mathcal A})$ where the $M_n({\mathcal A})$ embeds into $M_{n+1}({\mathcal A})$ block-diagonally as $M_n({\mathcal A}) \oplus 0$.

An idempotent *e* in an algebra ${\mathcal A}$ is said to be *properly infinite* if there are orthogonal idempotents $e_1, e_2$ in $e{\mathcal A}e$ such that $e_1 \sim e\sim e_2$. Equivalently, *e* is properly infinite if $e \gtrsim e \oplus e$. A (nonzero) unital algebra ${\mathcal A}$ is said to be *properly infinite* in case $\mathbb {1}$ is a properly infinite idempotent.

As an application of the dichotomy shown in Theorem 4.6, we present a method of producing non-algebraically amenable $\mathbb {K}$-algebras:

### Corollary 4.9

A properly infinite unital $\mathbb {K}$-algebra is not algebraically amenable.

### Proof

If ${\mathcal A}$ is properly infinite, it contains elements $u,v,u',v'$ satisfying the relations

$$\begin{aligned} u u' = v v' = \mathbb {1}_{{\mathcal A}}, \ v u' = 0=uv'. \end{aligned}$$

Suppose that there exists a $\{u,u',v,v'\}$-invariant dimension measure on ${\mathcal A}$ (associated to some basis). Notice that the first set of identities imply that $u'|_{\mathcal A}$ and $v'|_{\mathcal A}$ are injective. Thus by invariance, we have

$$\begin{aligned} 1= \mu ({\mathcal A}) \le \mu (u'{\mathcal A}) \le 1, \end{aligned}$$

which implies $\mu (u'{\mathcal A}) = \mu ({\mathcal A}) = 1$, and similarly $\mu (v'{\mathcal A}) = \mu ({\mathcal A}) = 1$. On the other hand, for any $a, b \in {\mathcal A}$ with $u'a = v' b$, we have $b = v v' b = v u' a = 0$ by the second identity. It follows that $u' {\mathcal A}\cap v' {\mathcal A}= 0$, and thus $\mu (u' {\mathcal A}+ v' {\mathcal A}) \ge \mu (u'{\mathcal A}) + \mu (v'{\mathcal A}) = 2$, which is an impossible value for $\mu$. This proves our claim. $\square$

## Leavitt algebras and Leavitt path algberas

In this section we study the amenability of Leavitt algebras and Leavitt path algebras (see below for the specific definitions). Classical Leavitt algebras were invented by Leavitt ([42, 43]) to provide universal examples of algebras without the invariant basis number property. As such, they cannot be algebraically amenable, by a result of Elek [32, Corollary 3.1(1)]. Leavitt path algebras provide a wide generalization of classical Leavitt algebras, in much the same way as graph $C^*$-algebras generalize Cuntz algebras (see e.g. [48] for an introduction to the theory of graph $C^*$-algebras).

### Leavitt algebras

Extending results by Aljadeff and Rosset [6] and Rowen [50], Elek proved in [32] that any finitely generated unital algebraically amenable $\mathbb {K}$-algebra ${\mathcal A}$ has the *Invariant Basis Number* (IBN) property, that is, any finitely generated free ${\mathcal A}$-module has a well-defined rank. This is equivalent to the condition

$$\begin{aligned} {\mathcal A}^n\cong {\mathcal A}^m \text { as left } {\mathcal A}\text {-modules} \implies n=m , \end{aligned}$$

for any positive integers *n*, *m*. We will use the observation in Corollary 4.9 to obtain a proof of the IBN property of general unital amenable algebras.

#### Definition 5.1

Let $\mathbb {K}$ be a field.

(i) Let *n*, *m* be integers such that $1\le m< n$. Then the Leavitt algebra $L(m,n)= L_\mathbb {K}(m,n)$ is the algebra generated by elements $X_{ij}$ and $Y_{ji}$, for $i=1,\ldots ,m$ and $j=1,\ldots ,n$, such that $XY = \mathbb {1}_m$ and $YX= \mathbb {1}_n$, where *X* denotes the $m\times n$ matrix $(X_{ij})$ and *Y* denotes the $n\times m$ matrix $(Y_{ji})$.
(ii) The algebra $L_{\infty }= L_{\mathbb {K}, \infty }$ is the unital algebra generated by $x_1,y_1,x_2, y_2, \ldots$ subject to the relations $y_jx_i = \delta _{i,j} \mathbb {1}$.

The algebras *L*(*m*, *n*) are simple if and only if $m=1$ [43, Theorems 2 and 3]. The algebra $L_{\infty }$ is simple [10, Theorem 4.3].

The following is well-known (cf. [2] or [42]):

#### Proposition 5.2

Let ${\mathcal A}$ be a (nonzero) unital algebra over a field $\mathbb {K}$.

1. ${\mathcal A}$ does not satisfy the IBN property if and only if there is a unital homomorphism $L(m,n) \rightarrow {\mathcal A}$ for some $1\le m < n$.
2. ${\mathcal A}$ is properly infinite if and only if there is a unital embedding $L_{\infty }\rightarrow {\mathcal A}$.

#### Proof

(1) By definition, if an algebra ${\mathcal A}$ does not have the IBN property, then there are *m*, *n* with $1\le m <n$ such that ${\mathcal A}^m \cong {\mathcal A}^n$, and this isomorphism of free modules will be implemented by matrices $X'\in M_{m\times n}({\mathcal A})$ and $Y' \in M_{n\times m} ({\mathcal A})$ such that $X'Y'=I_m$ and $Y'X'= I_n$. We thus obtain a unital homomorphism $L(m,n) \rightarrow {\mathcal A}$. The converse is trivial.

(2) If ${\mathcal A}$ is properly infinite, we may inductively find an infinite sequence $e_1, e_2,\ldots$ of mutually orthogonal idempotents such that $e_i\sim 1$ for all *i*. This enables us to define a homomorphism $L_{\infty } \rightarrow {\mathcal A}$ which is injective because $L_{\infty }$ is simple. The converse is obvious.$\square$

Note that $L_{\infty }$ is properly infinite but does have the IBN property.

#### Proposition 5.3

If ${\mathcal A}$ is a unital algebraically amenable algebra, then ${\mathcal A}$ has the IBN property.

#### Proof

Suppose that ${\mathcal A}$ does not have the IBN property. Then there are integers *m*, *n* with $1\le m <n$ and there is a unital homomorphism $L(m,n) \rightarrow {\mathcal A}$. Now $M_n({\mathcal A})\cong M_m({\mathcal A})$ is properly infinite, so that by Corollary 4.9, $M_n({\mathcal A})$ is not algebraically amenable. If ${\mathcal A}$ were amenable then $M_n({\mathcal A}) \cong {\mathcal A}\otimes M_n(\mathbb {K})$ would be amenable too ([25, Proposition 4.3(2)]). Therefore ${\mathcal A}$ is not algebraically amenable, showing the result. $\square$

#### Corollary 5.4

A unital $\mathbb {K}$-algebra ${\mathcal A}$ that unitally contains the Leavitt algebra *L*(*m*, *n*) for some $1\le m < n$ is not algebraically amenable. $\square$

### Leavitt path algebras

In general, a non-algebraically amenable algebra need not be properly infinite, as the non-commutative free algebra shows. We now show that, within a certain class of algebras, the class of *Leavitt path algebras*, both properties are indeed equivalent. Note that this class of algebras includes the algebras *L*(1, *n*) and $L_{\infty }$ as distinguished members. (The algebras *L*(*m*, *n*), with $1<m<n$ are not included in the class of Leavitt path algebras, but they are Morita-equivalent to Leavitt path algebras associated to separated graphs [8].) We refer the reader to [2] and the references therein for more information about Leavitt path algebras.

We recall some definitions needed here.

#### Definition 5.5

A (directed) graph $E=(E^{0},E^{1},r,s)$ consists of two sets $E^{0}$ and $E^{1}$ together with range and source maps $r,s:E^{1}\rightarrow E^{0}$. The elements of $E^{0}$ are called *vertices* and the elements of $E^{1}$ *edges*.

A vertex *v* is called a *sink* if it emits no edges, that is, $s^{-1}(v)=\varnothing$, the empty set. The vertex *v* is called a *finite emitter* if $s^{-1}(v)$ is finite; otherwise it is an *infinite emitter*. A finite emitter which is not a sink is also called a *regular vertex*. For each $e\in E^{1}$, we call $e^{*}$ a *ghost edge*. We let $r(e^{*})$ denote *s*(*e*), and we let $s(e^{*})$ denote *r*(*e*).

The Leavitt path algebras are built on top of these directed graphs.

#### Definition 5.6

Given an arbitrary graph *E* and a field $\mathbb {K}$, the *Leavitt path* $\mathbb {K}$ *-algebra* $L_{\mathbb {K}}(E)$ (or simply *L*(*E*)) is defined to be the $\mathbb {K}$-algebra generated by a set $\{v:v\in E^{0}\}$ of pairwise orthogonal idempotents together with a set of variables $\{e,e^{*}:e\in E^{1}\}$ which satisfy the following conditions:

1. $$s(e)e=e=er(e)$$ for all $$e\in E^{1}$$.
2. $$r(e)e^{*}=e^{*}=e^{*}s(e)$$ for all $$e\in E^{1}$$.
3. (The “CK-1 relations”) For all $e,f\in E^{1}$, $e^{*}e=r(e)$ and $e^{*}f=0$ if $e\ne f$.
4. (The “CK-2 relations”) For every regular vertex $v\in E^{0}$,
  $$\begin{aligned} v=\sum _{e\in E^{1},s(e)=v}ee^{*}. \end{aligned}$$

In a sense, the definition of a Leavitt path algebra treats the graph as a dynamical system: its multiplication is based on the ways one can traverse the vertices of the graph via the edges. This naturally brings into the picture notions such as paths and cycles.

#### Definition 5.7

A *(finite) path* $\mu$ of length $n>0$ is a finite sequence of edges $\mu =e_{1}e_{2}\cdot \cdot \cdot e_{n}$ with $r(e_{i})=s(e_{i+1})$ for all $i=1,\cdot \cdot \cdot ,n-1$. In this case, $\mu ^{*}=e_{n}^{*}\cdot \cdot \cdot e_{2}^{*}e_{1}^{*}$ is the corresponding ghost path. The set of all vertices on the path $\mu$ is denoted by $\mu ^{0}$. Any vertex *v* is considered a path of length 0.

A non-trivial path $\mu$ $=e_{1}\dots e_{n}$ in *E* is *closed* if $r(e_{n} )=s(e_{1})$, in which case $\mu$ is said to be *based at* the vertex $s(e_{1})$. By cyclically permuting the edges of a closed path $\mu =e_{1}\dots e_{n}$, we obtain a closed path $e_{k}\dots e_{n}e_{1}\dots e_{k-1}$ based at the vertex $s(e_{k})$ for any $k = 1, \ldots , n$. A closed path $\mu$ as above is called *simple* provided it does not pass through its base more than once, i.e., $s(e_{i})\ne s(e_{1})$ for all $i=2,...,n$.

The closed path $\mu$ is called a *cycle based at* *v* if $s(e_1)=v$ and it does not pass through any of its vertices twice, that is, if $s(e_{i})\ne s(e_{j})$ whenever $i\ne j$. A nontrivial cyclic permutation of a cycle based at a vertex *v* is then a cycle based at a different vertex. Cyclic permutation thus induces an equivalence relation on the set of all cycles based at vertices. An equivalence class of it is called a *cycle*. Note that it is meaningful to talk about the set of vertices of a cycle, which we denote by $c^0$. A cycle *c* is called an *exclusive cycle* if it is disjoint with every other cycle; equivalently, no vertex *v* on *c* is the base of a different cycle other than the cyclic permutation of *c* based at *v*.

The following lemma was shown in the row-finite case in [13, Lemma 7.3]. We include the identical proof for completeness.

#### Lemma 5.8

Let *E* be an arbitrary graph and let $\mathbb {K}$ be a field. If $v\in E^0$ belongs to a non-exclusive cycle, then *v* is a properly infinite idempotent in $L_\mathbb {K}(E)$.

#### Proof

We would like to show that $v \gtrsim v \oplus v$. To this end, let $e_1\dots e_m$ and $f_1\dots f_n$ be two different closed simple paths in *E* based at *v*. Then there is some positive integer *t* such that $e_i=f_i$ for $i=1,\ldots ,t-1$ while $e_t\ne f_t$. Thus, we have $s(e_{t})= s(f_{t})$ but $e_{t} \ne f_{t}$. We observe

$$\begin{aligned} v = s(e_1) \gtrsim r(e_1) = s(e_2) \gtrsim \ldots \gtrsim r(e_{t-1}) = s(e_{t}), \end{aligned}$$

and similarly $r(e_t) \gtrsim r(e_m) = v$ and $r(f_t) \gtrsim r(f_n) = v$. Since $e_t e_t^*$ and $f_t f_t^*$ are two mutually orthogonal idempotents below $s(e_{t})$, we have

$$\begin{aligned} v \gtrsim s(e_{t}) \gtrsim e_t e_t^* \oplus f_t f_t^* \sim e_t^* e_t \oplus f_t^* f_t = r(e_t)\oplus r(f_t) \gtrsim v\oplus v. \end{aligned}$$

Therefore *v* is properly infinite. $\square$

Below we summarize some additional basic terminologies and properties for graphs and Leavitt path algebras. For this we follow the book in preparation [1].

#### Remark 5.9

Let *E* be a directed graph.

1. If there is a path from a vertex *u* to a vertex *v*, we write $u\ge v$. This defines a pre-order on $E^0$. As we have shown above, $u\ge v$ implies $u \gtrsim v$ in $L_{\mathbb {K}}(E)$. Since all vertices on a cycle are equivalent with regard to the pre-order $\ge$, it induces a pre-order on the set of all cycles, so that for any cycles $c_1$ and $c_2$, we have $c_1\ge c_2$ if and only if there is path from a vertex of $c_1$ to a vertex of $c_2$.
2. Let *C* be the set of all cycles in *E*. Let $C/{\sim }$ be the partially ordered set obtained by antisymmetrization of the pre-order $\le$ on *C*, so that $c\sim c'$ if and only if $c \le c'$ and $c'\le c$. Note that the exclusive cycles are precisely those cycles *c* such that $[c]= \{ c \}$, and that $C/{\sim }$ is a finite set if *E* has a finite number of vertices.
3. The Leavitt path algebra $L_\mathbb {K}(E)$ is unital if and only if $| E^0 | < \infty$, in which case the unit is given by $\sum _{v \in E^0} v$.
4. Every finite path $\mu = e_1 \cdots e_n$ induces the elements $\mu = e_1 \cdots e_n$ and $\mu ^{*}=e_{n}^{*}\cdots e_{1}^{*}$ in $L_{\mathbb {K}}(E)$. By a simple induction, we see that the Leavitt path algebra $L_\mathbb {K}(E)$ is linearly spanned by terms of the form $\lambda \rho ^*$, where $\lambda$ and $\rho$ are paths such that $r(\lambda )= r(\rho )$.
5. The graph *E* is called *acyclic* if it contains no cycle, and *finite* if both $E^0$ and $E^1$ are finite sets. A finite acyclic graph clearly contains finitely many paths. Thus by (4), we see that $L_{\mathbb {K}}(E)$ is finite-dimensional. In fact, in this case, $L_{\mathbb {K}}(E)$ is a finite direct sum of matrix algebras over $\mathbb {K}$ (cf., [2, Theorem 3.1]).
6. A subset *H* of $E^{0}$ is called *hereditary* if, whenever $v\in H$ and $w\in E^{0}$ satisfy $v\ge w$, then $w\in H$. A hereditary set is *saturated* if, for any regular vertex *v*, $r(s^{-1}(v))\subseteq H$ implies $v\in H$. For $X\subseteq E^0$, we denote by $\overline{X}$ the hereditary saturated closure of *X*. To compute $\overline{X}$, one can first compute the *tree* of *X*, $T(X) := \{ w\in E^0 : w\le v \text { for some } v\in X \}$, which is the smallest hereditary subset of $E^0$ containing *X*, and then, setting $\Lambda _0(T(X)) := T(X)$, compute inductively
  $$\begin{aligned} \Lambda _n(T(X)) := \{y\in E^0_{\mathrm {reg}} : r(s^{-1}(y))\subseteq \Lambda _{n-1}(T(X))\} \cup \Lambda _{n-1}(T(X)) \end{aligned}$$
  for $n = 1, 2 , \ldots$, where $E^0_{\mathrm {reg}}$ is the set of regular vertices. It is easy to see $\overline{X} = \bigcup _{n=0}^{\infty } \Lambda _n(T(X))$.
7. We shall use the following constructions from [52]. A *breaking vertex *of a hereditary saturated subset *H* is an infinite emitter $w\in E^{0}{\setminus } H$ with the property that $1\le |s^{-1}(w)\cap r^{-1}(E^{0}{\setminus }H)|<\infty$. The set of all breaking vertices of *H* is denoted by $B_{H}$. For any $v\in B_{H}$, we define an idempotent $v^{H} \in L_{\mathbb {K}}(E)$ by
  $$\begin{aligned} v^{H} := v-\sum _{s(e)=v,r(e)\notin H}ee^{*}. \end{aligned}$$
  Given a hereditary saturated subset *H* and a subset $S\subseteq B_{H}$, (*H*, *S*) is called an *admissible pair.* Given an admissible pair (*H*, *S*), *I*(*H*, *S*) denotes the ideal generated by $H\cup \{v^{H}:v\in S\}$. Then we have an isomorphism $L_{\mathbb {K}}(E)/I(H,S)\cong L_{\mathbb {K}} (E / (H,S))$. Here *E* / (*H*, *S*) is the *quotient graph* of *E* in which $(E / (H,S))^{0}=(E^{0}\backslash H)\cup \{v^{\prime }:v\in B_{H}\backslash S\}$ and $(E / (H,S))^{1}=\{e\in E^{1}:r(e)\notin H\}\cup \{e^{\prime }:e\in E^{1},r(e)\in B_{H}\backslash S\}$ and *r*, *s* are extended to $(E / (H,S))^{1}$ by setting $s(e^{\prime })=s(e)$ and $r(e^{\prime })=r(e)^{\prime }$. Thus when $S=B_{H}$, we can identify the quotient graph $E{\setminus }(H,B_{H})$ with the subgraph *E* / *H* of *E*, where $(E / H)^0= E^0{\setminus }H$ and $(E / H)^1= \{ e\in E^1 : r(e) \notin H \}$. It was shown in [52] that the graded ideals of $L_{\mathbb {K}}(E)$ are precisely the ideals of the form *I*(*H*, *S*) for some admissible pair (*H*, *S*), though we will not make use of this.
8. A subgraph $E'$ of *E* is called *full* if $(E')^1 = \{e \in E^{1}:s(e), r(e) \in (E')^0 \}$. For a subset $X \subset E^0$, we define a full subgraph *M*(*X*) so that
  $$\begin{aligned} M(X)^0=\{w\in E^{0}:w\ge v \text { for some } v \in X \}. \end{aligned}$$
  If $X = \{v\}$ for some $v \in E^0$, we also write $M(v) = M(\{v\})$. Also define
  $$\begin{aligned} H(v)=E^{0}{\setminus }M(v)^0, \end{aligned}$$
  which is hereditary by design. Note that any edge *e* is in a cycle if and only if $r(e) \notin H(s(e))$ if and only if $r(e) \in M(s(e))^0$. It follows that if *v* belongs to a cycle, then *H*(*v*) is a hereditary saturated subset of *E*. $\square$

#### Theorem 5.10

Let *E* be a nontrivial directed graph and let $\mathbb {K}$ be a field. Let *H* be the smallest hereditary saturated subset of $E^0$ that contains all the cycles of *E*. Order the vertices and the cycles by the preorder defined in Remark 5.9 (1). Then we have the following three sets of equivalent conditions:

- The following are equivalent:
  1. $L_\mathbb {K}(E)$ is not algebraically amenable.
  2. $E^0$ is finite, $E^0 {\setminus } H = \varnothing$, and every maximal cycle is non-exclusive.
  3. $L_\mathbb {K}(E)$ is unital and properly infinite
- The following are equivalent:
  (A2) $L_\mathbb {K}(E)$ is algebraically amenable but not properly algebraically amenable.
  (B2) $E^0$ is finite, *E* is not acyclic, $E^0 {\setminus } H$ consists of a nonzero number of finite emitters, and every maximal cycle is non-exclusive.
  (C2) $L_\mathbb {K}(E) = L_\mathbb {K}(E') \oplus L_\mathbb {K}(E'')$ for some directed graphs $E'$ and $E''$ such that $L_\mathbb {K}(E')$ has nonzero finite dimension and $L_\mathbb {K}(E'')$ is not algebraically amenable.
- The condition
  (A3) $L_\mathbb {K}(E)$ is properly algebraically amenable
  holds if and only if one or more of the following conditions hold:
  - *E* is acyclic;
  - $$E^0$$ is infinite;
  - $E^0 {{\setminus }} H$ contains at least one infinite emitter;
  - *E* has an exclusive maximal cycle.

#### Proof

Write (B3) for the inclusive disjunction (B3a)$\vee$(B3b)$\vee$(B3c)$\vee$(B3d). We first observe that it suffices to show (B1) $\Rightarrow$ (C1), (B2) $\Rightarrow$ (C2), and (B3) $\Rightarrow$ (A3). Indeed, by Corollary 4.9, we have (C1) $\Rightarrow$ (A1), while by Example 3.7 and Proposition 3.8, we have (C2) $\Rightarrow$ (A2). Notice that the three conditions (A1), (A2) and (A3) are mutually exclusive, while the three conditions (B1), (B2) and (B3) exhaust all possible situations. It thus follows from basic logic that the three converse implications also hold, i.e., we have the full cycles

- (B1) $$\Rightarrow $$ (C1) $$\Rightarrow $$ (A1) $$\Rightarrow $$ (B1),
- (B2) $$\Rightarrow $$ (C2) $$\Rightarrow $$ (A2) $$\Rightarrow $$ (B2), and
- (B3) $$\Rightarrow $$ (A3) $$\Rightarrow $$ (B3).

We proceed now with the proofs of the three essential implications we need.

(B1) $\Rightarrow$ (C1): The unitality of $L_\mathbb {K}(E)$ follows directly from the finiteness of $E^0$ by Remark 5.9(3). Now let $[c_1], \ldots , [c_n]$ be the maximal elements of $C/{\sim }$, and pick a vertex $v_i$ in each cycle $c_i$. Since each $c_i$ is non-exclusive, by Lemma 5.8, each $v_i$ is a properly infinite idempotent, that is, $v_i\oplus v_i \lesssim v_i$. Since $\mathbb {1}= \sum _{v\in E^0} v$, to show that $\mathbb {1}$ is properly infinite, it suffices to check that $v\lesssim p:= \sum _{i=1}^n v_i$ for all $v\in E^0$. Set $X= \{v_1,\ldots , v_n \}$. By our assumption, $E^0 = H = \overline{X}$ and $E^0$ is finite; thus there is some *k* such that $E^0 = \Lambda _k (T(X))$. We show by induction on $r \in \mathbb {N}_0$ that $v\lesssim p$ for all $v\in \Lambda _r (T(X))$. For $r=0$, we have that $v\in T(X)$ and thus $v\le v_i$ for some *i*, which implies that $v\lesssim v_i \le p$. If $v\in \Lambda _r(T(X)){\setminus } \Lambda _{r-1} (T(X))$, then *v* is a regular vertex and, for any $e\in s^{-1}(v)$, we have $r(e)\in \Lambda _{r-1}(T(X))$, and thus $r(e) \lesssim p$ by the induction hypothesis. Hence

$$\begin{aligned} v= \sum _{e\in s^{-1} (v)} ee^* \sim \bigoplus _{e\in s^{-1}(v)} r(e) \lesssim p ^{\oplus |s^{-1}(v)|} \lesssim p, \end{aligned}$$

because *p* is properly infinite. This shows that $v\lesssim p$ for all $v\in \Lambda _r(T(X))$, completing the induction step. Therefore $\mathbb {1} \oplus \mathbb {1} \lesssim \mathbb {1}$, i.e., $L_\mathbb {K}(E)$ is properly infinite.

(B2) $\Rightarrow$ (C2): Define $E' = E/H$ and $E'' = M(H)$ (cf., Remark 5.9(7) and (8)). It follows from the assumptions that $E'$ has finitely many vertices and edges while $B_H= \varnothing$. By our notation in Remark 5.9(7), $I(H,\varnothing )$ denotes the ideal of $L_\mathbb {K}(E)$ generated by $\{v :v \in H\}$. We claim that there is an isomorphism $L_\mathbb {K}(E'') \cong I(H,\varnothing )$. To see this, for each $v \in E^0$, we let $\mathcal {P}_{\mathrm {min}}(v,H)$ be the set of minimal finite paths from *v* into *H*, i.e.,

$$\begin{aligned} \mathcal {P}_{\mathrm {min}}(v,H)= & {} \{ \text {path } \mu = e_1 \cdots e_n :s(e_1) = v , ~ r(e_n) \in H, ~ s(e_k)\\&\notin H \text { for } k = 1, \ldots n \}. \end{aligned}$$

By convention, if $v \in H$, then $\mathcal {P}_{\mathrm {min}}(v,H) = \{ v \}$. Note that $\mathcal {P}_{\mathrm {min}}(v,H)$ is non-empty precisely when $v \in M(H)^0$. Since each vertex in $E^0{\setminus }H$ is regular, there are only finitely many edges that may appear in the paths in $\mathcal {P}_{\mathrm {min}}(v,H)$ for any $v \in E^0$. By minimality, these paths cannot contain cycles; thus the set $\mathcal {P}_{\mathrm {min}}(v,H)$ is finite for each $v \in E^0$. Also note that for any two different paths $\mu , \nu \in \mathcal {P}_{\mathrm {min}}(v,H)$, we have $\mu ^* \nu = 0$ in $L_\mathbb {K}(E)$. Thus we may define, for any $v \in E^0$, an idempotent

$$\begin{aligned} \widehat{v} = \sum _{\mu \in \mathcal {P}_{\mathrm {min}}(v,H)} \mu \mu ^* \in I(H,\varnothing ). \end{aligned}$$

We may readily verify by Definition 5.6 that the prescription

$$\begin{aligned} v \mapsto \widehat{v} \text { for } v \in (E'')^0 \text { and } e \mapsto \widehat{s(e)} \, e \, \widehat{r(e)} \text { for } e \in (E'')^1 \end{aligned}$$

defines a (non-unital) graded homomorphism $L_\mathbb {K}(E'') \hookrightarrow L_\mathbb {K}(E)$ with image in $I(H,\varnothing )$. This map is injective by [52, Theorem 4.8]. On the other hand, by [52, Lemma 5.6], we have

$$\begin{aligned} I(H,\varnothing ) =&~ \mathrm {span}(\{ \mu \nu ^* :\mu \text { and } \nu \text { are paths with } r(\mu ) = r(\nu ) \in H \}) \\ =&~ \mathrm {span} \left( \left\{ \big ( \widehat{s(\mu )} \cdot \mu \cdot \widehat{r(\mu )} ) ( \widehat{r(\nu )} \cdot \nu ^* \cdot \widehat{s(\nu )} \big ) :r(\mu ) = r(\nu ) \in H \right\} \right) , \end{aligned}$$

which shows that the image of the above embedding contains $I(H,\varnothing )$. Therefore we have an isomorphism $L_\mathbb {K}(E'') \cong I(H,\varnothing )$. (We point out that another way of realizing $I(H,\varnothing )$ as a Leavitt path algebra is by using the hedgehog graph, cf. [1, Definitions 2.5.16 and 2.5.20].) Since $(E'')^0$ is finite, we see that $I(H,\varnothing )$ is unital as an algebra, with unit $p=\sum _{v\in M(H)^0} \widehat{v}$. It follows that *p* is a central idempotent in $L_\mathbb {K}(E)$, and that

$$\begin{aligned} L_\mathbb {K}(E') = L_\mathbb {K}(E/H) \cong L_\mathbb {K}(E)/I(H,\varnothing ) = (1-p)L_\mathbb {K}(E), \end{aligned}$$

and thus

$$\begin{aligned} L_\mathbb {K}(E) \cong L_\mathbb {K}(E/H) \oplus I(H,\varnothing ) \cong L_\mathbb {K}(E') \oplus L_\mathbb {K}(E''). \end{aligned}$$

Since *E* / *H* is a finite graph with no cycle, by Remark 5.9(5), we see that $L_\mathbb {K}(E')$ has finite dimension. On the other hand, by our construction of the graph $E''$, it inherits all the maximal cycles of *E*, which are all non-exclusive, and $(E'')^0$ is equal to the smallest hereditary saturated subset (with respect to $E''$) containing all the cycles. Thus $E''$ satisfies (B1). Since we have already proved (B1) $\Rightarrow$ (C1) $\Rightarrow$ (A1), we conclude that $L_\mathbb {K}(E'')$ is not algebraically amenable.

(B3a)$\vee$(B3b)$\vee$(B3c)$\vee$(B3d) $\Rightarrow$ (A3): We first observe that when (B3a) holds and (B3b) fails, i.e., when *E* is finite and acyclic, Remark 5.9 (5) tells us that $L_\mathbb {K}(E)$ is finite dimensional and thus properly algebraically amenable.

Apart from this easy case, $L_\mathbb {K}(E)$ is always infinite-dimensional, so by Proposition 3.5, it suffices to show that, given any $\varepsilon >0$, any $N \in \mathbb {N}$, and any finite subset $\mathcal F$ of $L_\mathbb {K}(E)$, we can find an $(\mathcal {F}, \varepsilon )$-Følner subspace *W* in $L_\mathbb {K}(E)$ with $\dim (W) \ge N$. Since each element of $L_\mathbb {K}(E)$ is a linear combination of terms of the form $\lambda \rho ^*$, where $\lambda$ and $\rho$ are paths such that $r(\lambda )= r(\rho )$, without loss of generality we can assume that $\mathcal F$ consists of elements of this form, say $\mathcal F = \{ \lambda _1\rho _1^*, \ldots , \lambda _r \rho _r^* \}$.

First, we assume (B3b) holds, i.e., $E^0$ is infinite. Then we can find a subset $X \subset E^0$ with $|X | = N$ and $X \cap \{ s(\rho _1) , \ldots , s(\rho _r) \} = \varnothing$. Put $W = \mathrm {span}(X)$. It then follows that $\lambda _j \rho _j^* W = 0$ for $j = 1, \ldots , r$. Hence *W* is an $(\mathcal {F}, 0)$-Følner subspace with $\dim (W) \ge N$.

Next, we assume (B3c) holds but (B3b) fails, i.e. $E^0$ is finite and $E^0{\setminus } H$ contains at least one infinite emitter. Let *v* be a maximal element among all infinite emitters of $E^0{\setminus } H$. Then *M*(*v*) contains no cycle and includes only finitely many vertices with no infinite emitter, and thus it also has only finitely many edges. By Remark 5.9(5), there are only finitely many paths in *E* ending in *v*. Since $s_E^{-1} (v)$ is infinite, there is $Y \subset s_E^{-1} (v)$ such that $|Y| = N$ and any $e \in Y$ is not contained in any of the paths $\rho _i$, for $i = 1, \ldots , r$. Define *W* to be the linear span of the finite set

$$\begin{aligned} \{ \tau e \in L_\mathbb {K}(E) :\tau \text { is a path ending in } v , ~ e \in Y \}. \end{aligned}$$

Notice that $\dim (W) \ge |Y| = N$. We claim that $\lambda _i \rho _i^* W\subset W$ for $i = 1, \ldots , r$. Indeed, since *e* is not an edge in $\rho _i$, the only way that the product $(\lambda _i \rho _i^*)(\tau e )$ is nonzero is that $\tau = \rho _i \tau '$ for some path $\tau '$ ending in *v*, whence

$$\begin{aligned} (\lambda _i \rho _i^*)(\tau e ) = \lambda _i\tau ' e \in W . \end{aligned}$$

This shows our claim. Hence *W* is an $(\mathcal {F}, 0)$-Følner subspace with $\dim (W) \ge N$.

Finally, we assume (B3d) holds but both (B3b) and (B3c) fail, i.e., $E^0$ is finite, $E^0{\setminus }H$ consists of regular vertices, and there is an exclusive maximal cycle, which we denote by *c*. Let $v_0$ be a vertex in *c* and let $\mu _0$ be the representative of *c* based at $v_0$. The subgraph $M(v_0)$ of *E* has the unique cycle *c*, and every vertex in $M(v_0)$ connects to it via paths. We claim that every vertex $v \in M(v_0)^0$ is regular in $M(v_0)$. Indeed, by Remark 5.9(8), every vertex in *c* only emits one edge in $M(v_0)$. On the other hand, any $v \in M(v_0)^0 {\setminus } H$ is regular even in *E* by our assumption. It remains to show that any $v \in M(v_0)^0 \cap H {\setminus } c^0$ is regular. For this we let $X \subset H$ consist of all the vertices of maximal cycles of *E*. Then by Remark 5.9(6), $H = \overline{X} = \bigcup _{k=0}^\infty \Lambda _k(T(X))$. It is clear by the maximality of the cycles that $M(v_0)^0 \cap T(X) = c^0$. Hence for any $v \in M(v_0)^0 \cap H {\setminus } c^0$, there is some $k \in \mathbb {N}_0$ such that $v \in \Lambda _{k+1}(T(X)) {\setminus } \Lambda _{k}(T(X))$; thus *v* is a regular vertex (even in *E*) by the definition of $\Lambda _{k+1}(T(X))$. This proves the claim. Now for each $v \in E^0$, we let $\mathcal {P}_{\mathrm {min}}(v,v_0)$ be the set of minimal finite paths from *v* to $v_0$, i.e.,

$$\begin{aligned} \mathcal {P}_{\mathrm {min}}(v,v_0)= & {} \{ \text {path } \mu = e_1 \cdots e_n :s(e_1) = v , ~ r(e_n) = v_0, \\&s(e_k) \not = v_0 \text { for } k = 1, \ldots n \}, \end{aligned}$$

By convention, $\mathcal {P}_{\mathrm {min}}(v_0,v_0) = \{ v_0 \}$. Note that $\mathcal {P}_{\mathrm {min}}(v,v_0)$ is a subset of all paths in $M(v_0)$ for each $v \in E^0$ and is non-empty precisely when $v \in M(v_0)^0$. Since every vertex $v \in M(v_0)^0$ is regular in $M(v_0)$, there are only finitely many edges that may appear in the paths in $\mathcal {P}_{\mathrm {min}}(v,v_0)$ for any $v \in E^0$. By minimality, these paths cannot contain cycles; thus the set $\mathcal {P}_{\mathrm {min}}(v,v_0)$ is finite for each $v \in E^0$. Thus the union $\mathcal {P} = \bigcup _{v \in E^0} \mathcal {P}_{\mathrm {min}}(v,v_0)$ of all minimal paths ending in $v_0$ is also finite. Note that any path ending in $v_0$ can be written uniquely as $\gamma \mu _0^k$ for some $\gamma \in \mathcal {P}$ and $k \in \mathbb {N}_0$. For each $k \in \mathbb {N}_0$, define a linear subspace $W_k$ of $L_\mathbb {K}(E)$ by

$$\begin{aligned} W_k = \mathrm {span}( \{ \gamma \mu _0^k :\gamma \in \mathcal {P} \} ) \end{aligned}$$

Thus for any different $k,l \in \mathbb {N}_0$, we have $\dim (W_k) = |\mathcal {P}|$ and the collection of subspaces $\{ W_k \}$ is independent. Let $N_1 \in \mathbb {N}$ be such that $N_1 |\mu _0|$ is greater than the length of each path among $\lambda _1, \ldots , \lambda _r, \rho _1, \ldots , \rho _r$, where $|\mu _0|$ is the length of $\mu _0$. For any $j \in \{1, \ldots , r\}$, $\gamma \in \mathcal {P}$ and $k \in \mathbb {N}$ with $k \ge N_1$, we claim that

$$\begin{aligned} \lambda _j\rho _j^* \gamma \mu _0^k \in \sum _{l = k-N_1}^{k+N_1} W_l. \end{aligned}$$

Indeed, this is trivial when $\rho _j^* \gamma \mu _0^k = 0$. If $\rho _j^* \gamma \mu _0^k \not = 0$, since $|\gamma \mu _0^k| > |\rho _j|$, we have $\gamma \mu _0^k = \rho _j \tau$ for some path $\tau$ ending in $v_0$. Hence $\lambda _j\rho _j^* \gamma \mu _0^k = \lambda _j \tau = \theta \mu _0^l$ for some $\theta \in \mathcal {P}$ and $l \in \mathbb {N}$. If $|\gamma | > |\rho _j|$, then $s(\tau ) \notin c^0$ and thus $l = k$. Otherwise we have the estimates

$$\begin{aligned} k |\mu _0| - |\rho _j| \le l |\mu _0| \le k |\mu _0| + |\lambda _j|. \end{aligned}$$

In either case, we have $k-N_1 \le l \le k+N_1$. This proves the claim. Now let $N_2 \in \mathbb {N}_0$ be such that $N_2 > N + N_1$ and $\frac{2 N_1}{N_2-N_1} \le \varepsilon$, and define

$$\begin{aligned} W = \sum _{k=N_1 + 1}^{N_2} W_k. \end{aligned}$$

Then $\dim (W) = |\mathcal {P}| (N_2 - N_1) \ge N$ and for any $j \in \{1, \ldots , r\}$, we have

$$\begin{aligned} \frac{\dim (\lambda _j\rho _j^* W + W)}{\dim (W)} \le \frac{ \dim ( \sum _{k=1}^{N_2+N_1} W_k ) }{ \dim ( \sum _{k=N_1+1}^{N_2} W_k ) } = \frac{| \mathcal P | (N_2+ N_1) }{| \mathcal P | (N_2-N_1)} \le 1 +\varepsilon . \end{aligned}$$

Hence *W* is an $(\mathcal {F}, \varepsilon )$-Følner subspace with $\dim (W) \ge N$.

Therefore any of the conditions (B3a), (B3b), (B3c) and (B3d) implies that $L_\mathbb {K}(E)$ is properly algebraically amenable. $\square$

We highlight the following trivial consequence of Theorem 5.10:

#### Corollary 5.11

Let *E* be a graph with finitely many vertices and let $\mathbb {K}$ be a field. Then the (unital) Leavitt path algebra $L_\mathbb {K}(E)$ is not algebraically amenable if and only if it is properly infinite.

#### Remark 5.12

It is well-known ([32, Proposition 3.1]) that a finitely generated $\mathbb {K}$-algebra of subexponential growth is amenable. On the other hand, it has been shown in [5] that, for a finite graph *E*, the Leavitt path algebra $L_{\mathbb {K}}(E)$ either has exponential growth or has polynomially bounded growth. Moreover, by [5, Theorem 5 (1)], $L_{\mathbb {K}}(E)$ has polynomially bounded growth if and only if every cycle of *E* is an exclusive cycle, and in this case a precise formula for the Gelfand–Kirillov dimension of $L_{\mathbb {K}}(E)$ is obtained ([5, Theorem 5 (2)]). Comparing this with Theorem 5.10, we see that there are finite graphs such that $L_{\mathbb {K}}(E)$ is algebraically amenable and has exponential growth (just consider the graph *E* of Example 5.15).

Since $L_\mathbb {K}(E)$ admits an involution (see for instance [52]), left and right amenability is equivalent for these algebras. Moreover the above proof shows that we can “localize” amenability in certain parts of the graph (in analogy with the metric space situation, cf., Sect. 2.1). We provide a simple example that shows that the situation is quite different when we consider the usual path algebras.

#### Definition 5.13

Given an arbitrary graph *E* and a field $\mathbb {K}$, the *path* $\mathbb {K}$ *-algebra* $\mathbb {K} E$ is defined to be the $\mathbb {K}$-algebra generated by a set $\{v:v\in E^{0}\}$ of pairwise orthogonal idempotents together with a set of variables $\{e:e\in E^{1}\}$ which satisfy $s(e)e=e=er(e)$ for all $e\in E^{1}$.

In other words, the path algebra is linearly spanned by all paths in *E*, with the multiplication given by concatenation of paths (or zero if two paths cannot be concatenated).

#### Example 5.14

Let *E* be the following graph:Let ${\mathcal A}$ be the corresponding path algebra $\mathbb {K}E$. We claim that ${\mathcal A}$ is left properly algebraically amenable but not right algebraically amenable.

To this end, we first observe, by checking on all paths in *E*, that for any $a \in {\mathcal A}$, we have $a v = vav = \kappa v$ for some $\kappa \in \mathbb {K}$, while $wa = waw$ and $vaw = xbw$ for some $b \in w{\mathcal A}w$. Since $v + w = \mathbb {1}$, we have the linear decomposition

$$\begin{aligned} {\mathcal A}= w {\mathcal A}w \oplus v {\mathcal A}w \oplus v {\mathcal A}v = w {\mathcal A}w \oplus x {\mathcal A}w \oplus \mathbb {K}v. \end{aligned}$$

Define the following linear maps:

$$\begin{aligned} \lambda :&~ {\mathcal A}\rightarrow w {\mathcal A}= w {\mathcal A}w ,&a \mapsto wa; \\ \rho :&~ {\mathcal A}\rightarrow {\mathcal A}w ,&a \mapsto aw; \\ \phi :&~ w {\mathcal A}w \rightarrow x {\mathcal A}w ,&a \mapsto x a. \end{aligned}$$

Then $\lambda$ and $\rho$ are surjections with kernels $v{\mathcal A}$ and ${\mathcal A}v$ ($= \mathbb {K}v$), respectively, while $\phi$ is a bijection. Also observe that the subalgebra $w {\mathcal A}w$ is isomorphic to the free algebra on two generators, and hence not algebraically amenable as it cannot carry an invariant dimension measure. In particular, both $w {\mathcal A}w$ and $x {\mathcal A}w$ have countably infinite dimension.

To see that ${\mathcal A}$ is left properly algebraically amenable, we choose an arbitrarily large finite-dimensional subspace *W* of $x {\mathcal A}w$ and note that ${\mathcal A}W = {\mathcal A}(v W ) = \mathbb {K}v W = W$, i.e., *W* is an $({\mathcal A}, 0)$-Følner subspace.

It remains to show that ${\mathcal A}$ is not right algebraically amenable. Since $w {\mathcal A}w$ is not algebraically amenable, by Lemma 4.3, there exists a finite subset ${\mathcal F}_0 \subset w {\mathcal A}w$ such that for any finite-dimensional subspace $W \subset w {\mathcal A}w$, we have $\dim (W \mathcal F _0 + W )\ge 3 \dim (W)$. Without loss of generality, we may assume $w \in {\mathcal F}_0$. Now define

$$\begin{aligned} {\mathcal F}= {\mathcal F}_0 \cup \{ x, v \}. \end{aligned}$$

Given an arbitrary nontrivial finite-dimensional subspace $W \subset {\mathcal A}$, we would like to show that $\dim (W \mathcal F + W )\ge 2 \dim (W)$.

First, if $W = \mathbb {K}v$, then $W \mathcal F + W = \mathbb {K}x \oplus \mathbb {K}v$, which has dimension 2, as desired. Now if $W \not = \mathbb {K}v$, or equivalently, $W w \not = 0$, then notice that

$$\begin{aligned} \dim (W) =&~ \dim (\rho (W)) + \dim (\mathrm {ker}(\rho ) \cap W) \\ =&~ \dim (W w) + \dim ( \mathbb {K}v \cap W) \\ =&~ \dim (\lambda (W w)) + \dim (\mathrm {ker}(\lambda ) \cap W w) + \dim ( \mathbb {K}v \cap W) \\ =&~ \dim (w W w) + \dim ( v {\mathcal A}\cap W w) + \dim ( \mathbb {K}v \cap W) \\ =&~ \dim (w W w) + \dim ( x {\mathcal A}w \cap W w) + \dim ( \mathbb {K}v \cap W). \end{aligned}$$

Similarly, we have

$$\begin{aligned} \dim (W {\mathcal F}+ W) =&~ \dim ( W {\mathcal F}_0 + Wx + W v + W) \\ =&~ \dim (w ( W {\mathcal F}_0 + Wx + W v + W ) w ) \\&+ \dim ( x {\mathcal A}w \cap ( W {\mathcal F}_0 + Wx + W v + W) w) \\&+ \dim ( \mathbb {K}v \cap ( W {\mathcal F}_0 + Wx + W v + W)) \\ =&~ \dim (w W w {\mathcal F}_0 ) + \dim ( x {\mathcal A}w \cap ( W w {\mathcal F}_0 + W x ) ) + \dim ( v W v) \\ \ge&~ \dim (w W w {\mathcal F}_0 ) + \dim ( ( x {\mathcal A}w \cap W w ) {\mathcal F}_0 ) + \dim ( v W v ) \\ =&~ \dim (w W w {\mathcal F}_0 ) + \dim ( \phi ^{-1} ( x {\mathcal A}w \cap W w ) {\mathcal F}_0 ) + \dim ( v W v) \\ \ge&~ 3 \dim (w W w) + 3 \dim (\phi ^{-1} ( x {\mathcal A}w \cap W w )) + \dim ( v W v) \\ =&~ 3 \dim (w W w) + 3 \dim ( x {\mathcal A}w \cap W w ) + \dim ( v W v) \\ =&~ 3 \dim (W w) + \dim (v W v). \end{aligned}$$

Here we used the fact that $\phi$ is a bijection and preserves multiplication from the right. Depending on whether $v \in W$ and whether $Wv = 0$, the pair $(\dim (v W v) , \dim ( \mathbb {K}v \cap W) )$ may take value among (0, 0), (1, 0) and (1, 1). In any case, since $\dim (W w) \ge 1$ by our assumption, we have

$$\begin{aligned} \frac{\dim (W {\mathcal F}+ W)}{ \dim (W) } \ge \frac{ 3 \dim (W w) + \dim (v W v) }{ \dim (W w) + \dim ( \mathbb {K}v \cap W) } \ge \frac{3 \dim (W w)+1}{ \dim (W w) + 1} \ge 2 \end{aligned}$$

as desired. Therefore ${\mathcal A}$ is not algebraically amenable. $\square$

The next example is similar to the above. It shows that having a maximal exclusive cycle is not enough to guarantee the (right) amenability of path algebras (compare with Theorem 5.10).

#### Example 5.15

Let *E* be the following graph:Here we also have that the path algebra $\mathcal A : = \mathbb {K}E$ is left properly algebraically amenable but not right algebraically amenable, despite the existence of an exclusive maximal cycle. Since the proof is similar to the one in the previous example, we only give a sketch, leaving the details to the reader.

In this case, we have a linear decomposition

$$\begin{aligned} \mathcal A = w\mathcal A w \oplus v\mathcal A v \oplus v\mathcal A w \cong w\mathcal A w \oplus \mathbb {K}[t] v \oplus x\mathcal A w \oplus tx\mathcal A w \oplus t^2 x\mathcal A w \oplus \cdots . \end{aligned}$$

For the left algebraic amenability, we can use a proper Følner net inside $\mathbb {K}[t]v$. On the other hand, for the right algebraic non-amenability, we again take ${\mathcal F}_0 \subset w {\mathcal A}w$ as in the previous example and set ${\mathcal F}= {\mathcal F}_0 \cup \{ x, v \}$. Given an arbitrary finite-dimensional subspace $W \subset {\mathcal A}$, if $\dim ({\mathcal A}v \cap W) \ge \frac{3}{5} \dim (W)$, then

$$\begin{aligned} \dim (W {\mathcal F}) \ge \dim ( ({\mathcal A}v \cap W ) \cdot \{x,v\} ) = 2 \dim ({\mathcal A}v \cap W ) \ge \frac{6}{5} \dim (W). \end{aligned}$$

Otherwise, we have $\dim (W w) = \dim (W / ({\mathcal A}v \cap W) ) = \dim (W) - \dim ({\mathcal A}v \cap W) > \frac{2}{5} \dim (W)$. Note that *Ww* is contained in a finitely generated free right $w\mathcal A w$-module $w\mathcal A w \oplus x\mathcal A w \oplus tx\mathcal A w \oplus t^2 x\mathcal Aw \oplus \cdots \oplus t^k x\mathcal Aw$ for some $k \in \mathbb {N}_0$. Thus by iterating the argument we used in the previous example (where we had $Ww \subset w\mathcal A w \oplus x\mathcal A w$), we can show

$$\begin{aligned} \dim (W {\mathcal F}) \ge \dim ( W w \cdot {\mathcal F}_0 ) \ge 3 \dim ( W w ) > \frac{6}{5} \dim (W). \end{aligned}$$

Thus ${\mathcal A}$ is not right algebraically amenable. $\square$

## Translation algebras on coarse spaces

To conclude we will illustrate the close relation between amenability for metric spaces and algebraic amenability for $\mathbb {K}$-algebras, in view of the natural bridge between the two settings—the construction of translation algebras (see, e.g., [49, Chapter 4]). Let us recall this construction.

Let (*X*, *d*) be a locally finite extended metric space as in Sect. 2 and $\mathbb {K}$ an arbitrary field. We denote by $\mathbb {K}[X]$ the $\mathbb {K}$-linear space generated by the basis *X*, and by $\mathrm {End}_{\mathbb {K}}(\mathbb {K}[X])$ the algebra of $\mathbb {K}$-linear endomorphism of $\mathbb {K}[X]$. For the sake of clarity, we denote by $\delta _x$ the basis element of $\mathbb {K}[X]$ corresponding to a point $x \in X$. We also sometimes think of an element $T \in \mathrm {End}_{\mathbb {K}}(\mathbb {K}[X])$ as a matrix indexed by *X*, and define $T_{xy} \in \mathbb {K}$ as its entry at $(x,y ) \in X \times X$, so that $T(\delta _y) = \sum _{x\in X} T_{xy} \delta _x$ for any $y \in X$.

For any partial translation *t* on *X* (cf. Definition 2.7), we define $V_t \in \mathrm {End}_{\mathbb {K}}(\mathbb {K}[X])$ by

6.1 $$\begin{aligned} V_t(\delta _x):= {\left\{ \begin{array}{ll} \delta _{t(x)} &{} \text {if}\ x \in \mathrm {dom}(t)\\ 0 &{} \text {if}\ x \notin \mathrm {dom}(t). \end{array}\right. } \end{aligned}$$

Note that for any two partial translations *t* and $t'$ on *X*, we have $V_t V_{t'} = V_{t \circ t'}$. In other words, $t \mapsto V_t$ gives a representation of the semigroup $\mathrm {PT}(X)$.

### Definition 6.1

The *translation* $\mathbb {K}$ *-algebra* $\mathbb {K}_\mathrm {u}(X)$ is the (unital) $\mathbb {K}$-subalgebra of $\mathrm {End}_{\mathbb {K}}(\mathbb {K}[X])$ generated by $V_t$ for all the partial translations *t* on *X*.

Any subset $A \subset X$ gives rise to an idempotent $V_{\mathrm {Id}_A}$ in $\mathbb {K}_\mathrm {u}(X)$, where $\mathrm {Id}_A$ is the identity map on *A*. For the sake of simplicity, we denote this idempotent by $P_A$. In particular, $P_X$ is equal to the unit of $\mathrm {End}_{\mathbb {K}}(\mathbb {K}[X])$. Note that we have the identities

$$\begin{aligned} V_{t^{-1}} V_t=P_{\mathrm {dom}(t)} \quad \mathrm {and} \quad V_t V_{t^{-1}}=P_{\mathrm {ran}(t)} \end{aligned}$$

for any partial translation *t* on *X*. Moreover, any element in $\mathbb {K}_\mathrm {u}(X)$ can be linearly spanned by the generators $V_t$.

Given a matrix $T \in \mathrm {End}_{\mathbb {K}}(\mathbb {K}[X])$ it is useful to consider its propagation as defined by

$$\begin{aligned} p(T):=\sup \Big \{d(x,y):x,y\in X\quad \mathrm {and}\quad T_{xy}\ne 0\Big \}. \end{aligned}$$

It is clear that every element in the translation $\mathbb {K}$-algebra has finite propagation and that for any $A\subset X$ we have $p(P_A)=0$.

### Remark 6.2

One can easily see that whenever we have a decomposition of an extended metric space *X* into a finite disjoint union $X_1 \sqcup \ldots \sqcup X_n$ with infinite distance between each pair of subspaces, then the associated idempotents $P_{X_1} , \ldots , P_{X_n}$ are central and mutually orthogonal, and add up to the unit, which induces a direct sum decomposition

$$\begin{aligned} \mathbb {K}_\mathrm {u}(X) \cong \bigoplus _{i = 1} ^n \mathbb {K}_\mathrm {u}(X_i). \end{aligned}$$

### Theorem 6.3

Let (*X*, *d*) be a locally finite extended metric space and let $\mathbb {K}_\mathrm {u}(X)$ be its translation $\mathbb {K}$-algebra. Let $n \ge 2$ be a natural number. Then the following conditions are equivalent:

1. (*X*, *d*) is amenable.
2. $\mathbb {K}_\mathrm {u}(X)$ is algebraically amenable.
3. $\mathbb {K}_\mathrm {u}(X)$ is not properly infinite.
4. $\mathbb {K}_\mathrm {u}(X)$ does not contain the Leavitt algebra $L_{\mathbb {K}}(1,n)$ as a unital $\mathbb {K}$-subalgebra.

### Proof

(1) $\Rightarrow$ (2): Consider $\varepsilon > 0$ and a finite set $\mathcal {F}\subset \mathbb {K}_\mathrm {u}(X)$. We may assume that any element in ${\mathcal F}$ has propagation at most $R>0$. Since (*X*, *d*) is amenable, and using the conventions in Definition 2.1, there exists a (finite, non-empty set) . We first show that we may assume that *F* is contained in a single coarse component of *X*. Indeed, write $F= \bigsqcup _{i=1}^N F_i$, where $F_i$, $i=1,\dots ,N$, are the coarse components of *F*. We then have $\sum _{i=1}^N |\partial _R(F_i)| / |F| \le \varepsilon$. Suppose that $|\partial _R(F_i)| / |F_i| > \varepsilon$ for all *i*. Then we have

$$\begin{aligned}\sum _{i=1}^N \frac{|\partial _R(F_i)|}{|F|} = \sum _{i=1}^N \frac{|F_i|}{|F|} \cdot \frac{|\partial _R(F_i)|}{|F_i|} >\Big ( \sum _{i=1}^N \frac{|F_i|}{|F|} \Big ) \varepsilon = \varepsilon , \end{aligned}$$

a contradiction. Thus, by replacing *F* with some of the its coarse components, we may assume that *F* is contained in a coarse component of *X*. It follows from the definition of propagation that whenever $d(Y, Y') > R$, then any $T\in {\mathcal F}$ satisfies $P_Y T P_{Y'} = 0$. Now we define the following linear subspace in $\mathbb {K}_\mathrm {u}(X)$ (in fact a subalgebra),

$$\begin{aligned} W := P_F \mathbb {K}_\mathrm {u}(X) P_F \subset \mathbb {K}_\mathrm {u}(X), \end{aligned}$$

which satisfies that $\dim W = |F|^2$.

We analyze next for any $T\in \mathcal {F}$ the subspace *TW* as follows. To simplify expressions we will use the standard notation for the commutator of two operators: $[T, B] := TB-BT$. Using the notation of *R*-boundaries and neighborhoods of Sect. 2 we have

$$\begin{aligned} \mathbb {1} = P_F + P_{X {\setminus } F} = (P_{N^-_{R}F} + P_{\partial ^-_R F}) + (P_{\partial ^+_R F} + P_{X {\setminus } N^+_R F} ) \end{aligned}$$

as well as

$$\begin{aligned} P_{N_{R}^-F} T P_{X {\setminus } F} = P_{X {\setminus } F} T P_{N_{R}^-F} = P_{X {\setminus } N_R^+ F} T P_F = P_F T P_{X {\setminus } N_R^+ F} = 0. \end{aligned}$$

Then we have

$$\begin{aligned} T P_F =&\ (P_F + P_{\partial ^+_R F} + P_{X {\setminus } N_R^+ F} ) T P_F \\ =&\ P_F T P_F + P_{\partial ^+_R F} T (P_{N_{R}^-F} + P_{\partial ^-_R F}) + 0 \\ =&\ P_F T P_F + 0 + P_{\partial ^+_R F} T P_{\partial ^-_R F}, \end{aligned}$$

and similarly

$$\begin{aligned} P_F T = P_F T P_F + P_{\partial ^-_R F} T P_{\partial ^+_R F}. \end{aligned}$$

Hence

6.2 $$\begin{aligned}{}[T, P_F] = P_{\partial ^+_R F} T P_{\partial ^-_R F} - P_{\partial ^-_R F} T P_{\partial ^+_R F}, \end{aligned}$$

and

6.3 $$\begin{aligned} T W= & {} \{T\, P_F B P_F \,:\, B\in \mathbb {K}_\mathrm {u}(X)\} \nonumber \\= & {} \{P_F TB P_F + [T,P_F]\,BP_F \, :\, B\in \mathbb {K}_\mathrm {u}(X)\} \nonumber \\= & {} \{P_F TB P_F + P_{\partial ^+_R F} T P_{\partial ^-_R F} \,BP_F - P_{\partial ^-_R F} T P_{\partial ^+_R F} \,BP_F \, :\, B\in \mathbb {K}_\mathrm {u}(X)\}\nonumber \\ \end{aligned}$$

6.4 $$\begin{aligned}\subseteq & {} W + P_{\partial ^+_R F} \mathbb {K}_\mathrm {u}(X) P_{F} + P_{\partial ^-_R F} \mathbb {K}_\mathrm {u}(X) P_{F}. \end{aligned}$$

Therefore we have the following estimates for any $T\in {\mathcal F}$:

$$\begin{aligned} \frac{\dim (TW +W )}{\dim (W )}\le & {} \frac{\dim (W ) + \dim (P_{\partial ^+_R F} \mathbb {K}_\mathrm {u}(X) P_{F} ) + \dim ( P_{\partial ^-_R F} \mathbb {K}_\mathrm {u}(X) P_{F})}{\dim (W )} \\\le & {} 1+ \frac{|F|\,|\partial ^+_R F|+|F|\,|\partial ^-_R F|}{|F|^2} \\= & {} 1+\frac{ \, |\partial _R F| }{|F|} \;\le \; 1+ \varepsilon . \end{aligned}$$

This shows that $\mathbb {K}_\mathrm {u}(X)$ is algebraically amenable.

(2) $\Rightarrow$ (3): This implication follows from Corollary 4.9.

(3) $\Rightarrow$ (4): Suppose that for some $n\ge 2$ the Leavitt algebra *L*(1, *n*) unitally embeds into $\mathbb {C}_\mathrm {u}(X)$. Then, any two distinct pairs of generators $X_i,Y_i$, $X_j,Y_j$, $i\not =j$, of *L*(1, *n*) implement the proper infiniteness of $\mathbb {K}_\mathrm {u}(X)$.

(4) $\Rightarrow$ (1): Assume that (*X*, *d*) is not amenable. Then by Theorem 2.17 *X* is paradoxical, i.e., there is a partition $X=X_+\sqcup X_-$ and partial translations $t_{\pm }:X\rightarrow X_\pm$. The corresponding generators of the translation algebra $V_{t_\pm }$, $V_{t_\pm ^{-1}}$ satisfy

$$\begin{aligned} V_{t_+}V_{t_+^{-1}}+V_{t_-}V_{t_-^{-1}} =\mathbb {1},\quad V_{t_\pm ^{-1}}V_{t_\pm }=\mathbb {1}\quad \mathrm {and}\quad V_{t_\pm ^{-1}}V_{t_\mp }=0. \end{aligned}$$

This shows that *L*(1, 2) unitally embeds into the translation $\mathbb {K}$-algebra. The result then follows from the fact that *L*(1, *n*) unitally embeds into *L*(1, 2) (see [22, Theorem 4.1]). $\square$

We also have an analogous result for proper amenability. We will use the following terminology. Given two algebras $\mathcal A$ and $\mathcal B$, we say that $\mathcal A$ is a *finite-dimensional extension of* $\mathcal B$ in case there is a finite-dimensional two-sided ideal *I* of $\mathcal A$ such that $\mathcal A/ I \cong \mathcal B$.^4^

### Theorem 6.4

Let (*X*, *d*) be a locally finite extended metric space and let $\mathbb {K}_\mathrm {u}(X)$ be its translation $\mathbb {K}$-algebra. Let $n \ge 2$ be a natural number. Then the following conditions are equivalent:

1. (*X*, *d*) is properly amenable.
2. $\mathbb {K}_\mathrm {u}(X)$ is properly algebraically amenable.
3. $\mathbb {K}_\mathrm {u}(X)$ is not a finite-dimensional extension of a properly infinite $\mathbb {K}$-algebra.

### Proof

(1) $\Rightarrow$ (2): Assume that (*X*, *d*) is properly amenable and recall the proof of the implication (1) $\Rightarrow$ (2) in Theorem 6.3. For $R > 0$, $\varepsilon >0$ and $N\in \mathbb {N}$, we can choose by Lemma 2.6 a (finite, non-empty) set  with $|F|\ge 2 N$. Let $F=\bigsqcup _{i\in I} F_i$ be the decomposition of *F* into its coarse components. Let

$$\begin{aligned}I' := \left\{ i \in I :\frac{|\partial _R F_i |}{|F_i|} \le \varepsilon \right\} \end{aligned}$$

and let $F' := \bigsqcup _{i\in I'} F_i$. We observe that $|F'| \ge \frac{1}{2} |F| \ge N$. Indeed, if this were not true, then

$$\begin{aligned}\frac{|\partial _R F |}{|F|} \ge \frac{\sum _{i \in I \setminus I'}|\partial _R F_i |}{|F|}> \frac{\sum _{i \in I \setminus I'} \varepsilon |F_i |}{|F|} = \frac{ \varepsilon | F \setminus F' |}{|F|} > \frac{\varepsilon \frac{1}{2} | F |}{|F|} = \frac{\varepsilon }{2} \; , \end{aligned}$$

a contradiction to . For each $i \in I'$, let $W_i := P_{F_i} \mathbb {K}_\mathrm {u}(X) P_{F_i}$. Then as in the proof of the implication (1) $\Rightarrow$ (2) in Theorem 6.3, we have $\dim W_i = |F_i|^2$ and for any *T* with propagation no more than *R*, we have $\dim (T W_i + W_i ) \le |F_i| (|F_i| + |\partial _R F_i|) \le |F_i|^2 (1 + \varepsilon )$. Hence if we let $W = \sum _{i \in I'} W_i$, we have

$$\begin{aligned} \dim (W) = \sum _{i \in I'} \dim (W_i ) = \sum _{i \in I'} |F_i|^2 \ge \sum _{i \in I'} |F_i| = |F'| \ge N \end{aligned}$$

and for any *T* with propagation no more than *R*

$$\begin{aligned} \frac{\dim (TW +W )}{\dim (W )} = \frac{\sum _{i \in I'} \dim (T W_i + W_i )}{\sum _{i \in I'} \dim (W_i )} \le \frac{\sum _{i \in I'} |F_i|^2 (1 + \varepsilon )}{\sum _{i \in I'} |F_i|^2} = 1 + \varepsilon \end{aligned}$$

Hence, by Proposition 3.5, we have that $\mathbb {K}_\mathrm {u}(X)$ is properly algebraically amenable.

(2) $\Rightarrow$ (3): Suppose that $\mathbb {K}_\mathrm {u}(X)$ is a finite-dimensional extension of a properly infinite $\mathbb {K}$-algebra, that is, there is a finite-dimensional two-sided ideal *I* of $\mathbb {K}_\mathrm {u}(X)$ such that $\mathbb {K}_\mathrm {u}(X) / I$ is properly infinite. By Corollary 4.9, $\mathbb {K}_\mathrm {u}(X) / I$ is not algebraically amenable, and thus not properly algebraically amenable, either. By Proposition 3.8, it follows that $\mathbb {K}_\mathrm {u}(X)$ is not properly algebraically amenable.

(3) $\Rightarrow$ (1): Assume that $\mathbb {K}_\mathrm {u}(X)$ is not a finite-dimensional extension of a properly infinite $\mathbb {K}$-algebra. In particular, itself is not properly infinite. Then Theorem 6.3 implies that (*X*, *d*) is amenable. Now suppose that (*X*, *d*) were *not* a properly amenable metric space. Corollary 2.20 shows that there would be a partition $X= Y_1\sqcup Y_2$, where $Y_1$ is a finite non-empty subset of *X*, $Y_2$ is non-amenable and $d(x,y)= \infty$ for $x\in Y_1$ and $y\in Y_2$. As in Remark 6.2, this would induce a direct sum decomposition $\mathbb {K}_\mathrm {u}(X) \cong \mathbb {K}_\mathrm {u}(Y_1)\oplus \mathbb {K}_\mathrm {u}(Y_2)$, with $\mathbb {K}_\mathrm {u}(Y_1)$ being finite-dimensional. In particular, $\mathbb {K}_\mathrm {u}(X)$ would be a finite-dimensional extension of $\mathbb {K}_\mathrm {u}(Y_2)$, the latter being properly infinite, again by Theorem 6.3. This would contradict our assumption. $\square$

## Footnotes

1 In fact, Elek’s original definition in [32] corresponds formally to our definition of proper algebraic amenability, instead of algebraic amenability. For general algebras with possible zero-divisors, we prefer to assign the term “algebraic amenability” to the concept without the exhaustion requirement because of its central role in Theorem 2.

2 Recall that a metric space is locally finite if and only if it is discrete and proper, the latter meaning that any closed ball is compact (see, e.g., [21, Section 5.5]). We avoid this terminology because we use the term “proper” in a different sense in this article.

3 It is also called *doubling condition* in the survey of Elek and Sós [35] and in [23].

4 This is in agreement with the non-universal convention of calling the algebra $\mathcal A$ above an extension of $\mathcal B$ by *I*.
